# Inducible knockout of *Clec16a* in mice results in sensory neurodegeneration

**DOI:** 10.1038/s41598-021-88895-0

**Published:** 2021-04-29

**Authors:** Heather S. Hain, Rahul Pandey, Marina Bakay, Bryan P. Strenkowski, Danielle Harrington, Micah Romer, William W. Motley, Jian Li, Eunjoo Lancaster, Lindsay Roth, Judith B. Grinspan, Steven S. Scherer, Hakon Hakonarson

**Affiliations:** 1 The Center for Applied Genomics, Children’s Hospital of Philadelphia, Philadelphia, PA 19104 USA; 2 Department of Neurology, The Perelman School of Medicine, University of Pennsylvania, Philadelphia, PA 19104 USA; 3 Department of Neurology, Children’s Hospital of Philadelphia, Philadelphia, PA 19104 USA; 4 Department of Pediatrics, The Perelman School of Medicine, University of Pennsylvania, Philadelphia, PA 19104 USA

**Keywords:** Neurodegeneration, Inflammation, Genetics, Immunology, Neuroscience, Neurology

## Abstract

CLEC16A has been shown to play a role in autophagy/mitophagy processes. Additionally, genetic variants in *CLEC16A* have been implicated in multiple autoimmune diseases. We generated an inducible whole-body knockout, *Clec16a*^ΔUBC^ mice, to investigate the loss of function of CLEC16A. The mice exhibited a neuronal phenotype including tremors and impaired gait that rapidly progressed to dystonic postures. Nerve conduction studies and pathological analysis revealed loss of sensory axons that are associated with this phenotype. Activated microglia and astrocytes were found in regions of the CNS. Several mitochondrial-related proteins were up- or down-regulated. Upregulation of interferon stimulated gene 15 (IGS15) were observed in neuronal tissues. CLEC16A expression inversely related to IGS15 expression. ISG15 may be the link between CLEC16A and downstream autoimmune, inflammatory processes. Our results demonstrate that a whole-body, inducible knockout of *Clec16a* in mice results in an inflammatory neurodegenerative phenotype resembling spinocerebellar ataxia.

## Introduction

Several studies have described a role of CLEC16A in autophagic processes, particularly an inhibitory role on autophagy induction^1–4^ and mitophagy regulation^5–7^. CLEC16A is a membrane-associated endosomal protein, E3 ubiquitin ligase, that forms a ubiquitin-dependent complex with NRDP1 and USP8^5^. Maintenance of the CLEC16A–NRDP1–USP8 mitophagy complex is necessary to sustain mitochondrial functions important for optimal cellular functions. Mitophagy, clearing of damaged or dysfunctional mitochondria, is crucial for mitochondrial quality control. Previously, we demonstrated the connection between CLEC16A and pancreatic ß cell function through mitophagy^7^. Loss of CLEC16A leads to an increase in the NRDP1 target PARKIN (PARK2), a master regulator of mitophagy^7^. We have found the loss of CLEC16A in immune cells results in dysregulated mitophagy and upregulated inflammatory cytokine response that increases the risk of autoimmunity^8^.

*CLEC16A* also is genetically linked with many distinct autoimmune disorders, including but not limited to type-1 diabetes, multiple sclerosis, systemic sclerosis, systemic lupus erythematosus, and rheumatoid arthritis^9–13^. Despite strong genetic evidence implicating *CLEC16A* in autoimmunity, this association remains unexplained mechanistically. CLEC16A may be interacting with other proteins to connect mitophagy/autophagy with autoinflammation/autoimmune processes. In preliminary studies, Tripartite motif 25 [TRIM25, aka estrogen-responsive finger protein (EFP)], an E3 ubiquitin, interferon stimulated gene 15 (ISG15) ligase, was identified as interacting with CLEC16A (Supplemental Table 1). ISG15, a ubiquitin-like modifier that inhibits polyubiquitination and is involved in a post-translational modification process called ISGylation, has a role in autophagy by conjugating with other proteins to control clearance of protein aggregates^14^. Activation of ISIG15 occurs after cell stress^15^, when neuronal damage is present^16–18^, and is hypothesized to be a potential cause of defective mitophagy in neurodegenerative diseases^19^. ISG15 and ISGylation are implicated in the control of mitochondrial OXPHOS and recycling in bone marrow-derived macrophages^20^. ISG15 conjugation to Parkin enhances its E3 ubiquitin ligase activity; this is an example of how ISGylation affects mitochondrial processes in neurodegeneration^21^. Free ISG15 stimulates the production of IFN-γ^22^. Because of its many roles in ubiquitination and inflammatory processes, we hypothesized ISG15 could be the link between CLEC16A and downstream autoimmune and inflammatory processes.

To better understand the role of CLEC16A in mitophagy and other autoimmune-inflammatory disorders besides diabetes, we created a *Clec16a* whole-body, inducible-knockout mouse model (*Clec16a*^ΔUBC^). *Clec16a*^ΔUBC/ΔUBC^ homozygote mice (constitutive knockout) are embryonic lethal based on unequal genotype distribution in heterozygous breeding crosses, therefore, an inducible model was utilized. In 2016, Redmann et al. described two constitutive *Clec16a* mutant mouse models (*Clec16a*^*GT/GT*^ and *Clec16a*^*CURT/CURT*^) with a neurodegenerative phenotype including Purkinje cell loss and locomotor deficits^1^. Because of the embryonic lethality of the constitutive *Clec16a*^ΔUBC/ΔUBC^ mice, we anticipated the severity of the phenotype may be greater in our inducible model than in the other constitutive mutants. In addition, the previous study only examined CNS areas where Clec16a is highly expressed, namely the cerebellar Purkinje cells and neurons of the deep cerebellar nuclei^23^.

In the present study, we describe a neurological phenotype in which *Clec16a*^ΔUBC^ mice develop severe neurologic dysfunction, including impaired gait and dystonic postures, in association with the degeneration of primary sensory axons. Astrogliosis and activated microglia are present in regions with axonopathy, and evidence of dysregulated autophagy and mitophagy in neural regions with and without axonopathy is observed. Alterations in mitochondrial-related proteins, ER stress markers, and IGS15 were observed in neuronal tissues. Hence, our *Clec16a*^ΔUBC^ mice are a suitable animal model of sensory neuron degeneration, and further illuminate a role for CLEC16A in inflammatory neurodegeneration.

## Results

### Rapidly progressive neurological disease in *Clec16a*^ΔUBC^ mice

**Figure 1:**
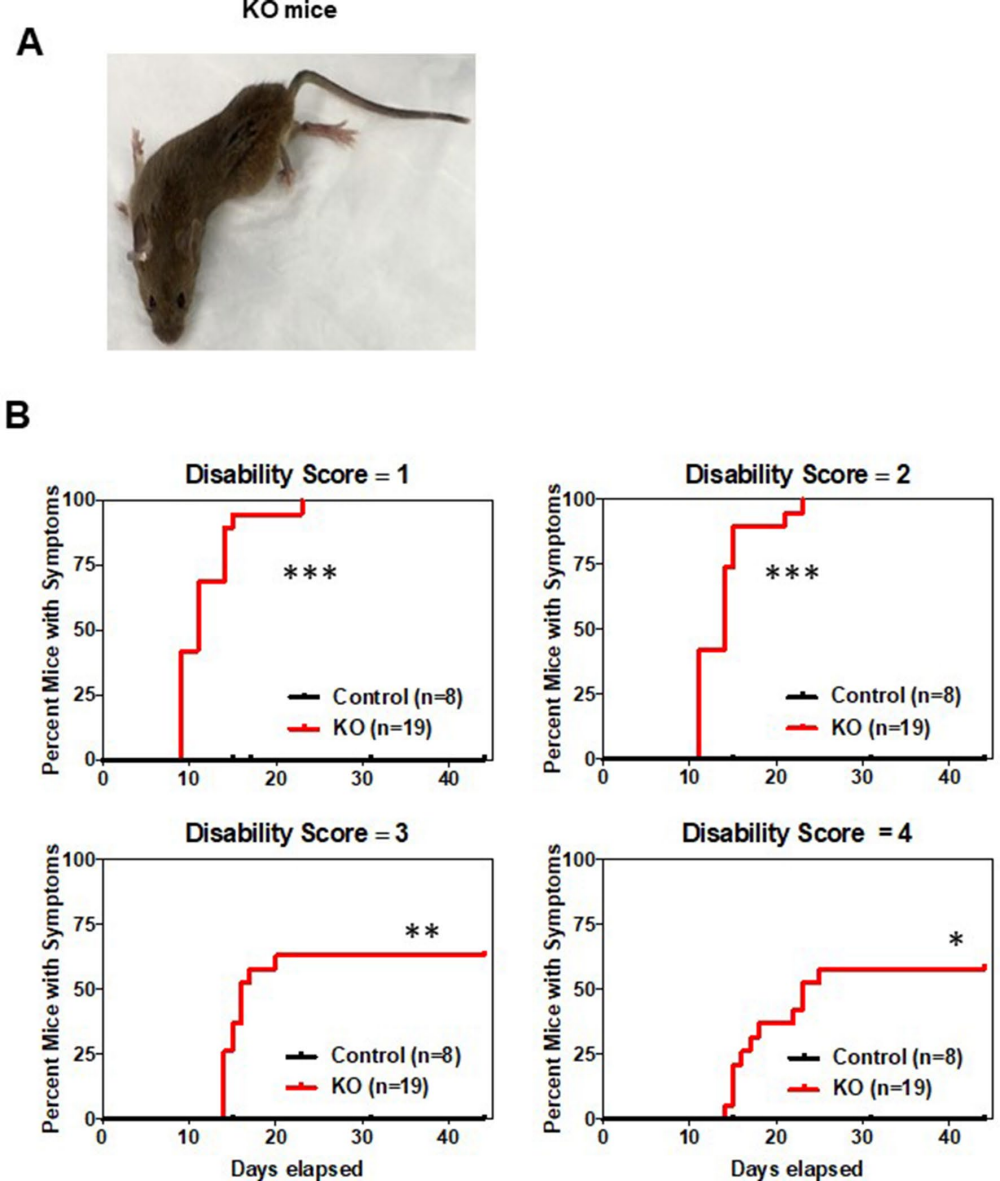
*Clec16a*^ΔUBC^ (whole-body, inducible knockout) mice exhibit neurological disability. (**A**) Image illustrating dystonic posturing in a representative ubiquitous, inducible KO (*Clec16a*^ΔUBC^) mouse around 15 days post induction. (**B**) Timeline to onset of disability (scores 1–4) in a cohort of control/*Clec16a*^loxP/loxP^ and KO/*Clec16a*^ΔUBC^ mice after treating P73–79 mice with tamoxifen for 4 successive days. Number of mice/group indicated on each graph; both sexes were used. *P < 0.05, **P < 0.01, ***P < 0.001 (Control vs. KO) for each disability score as analyzed by the Mantel–Cox test for survival curves.

Young adult *UBC-Cre-ER*^*T2*^*-Clec16a*^loxP/loxP^ mice were treated with tamoxifen for five successive days to generate adult *Clec16a* knockout (*Clec16a*^ΔUBC^) mice as described previously^8^. Prior to treatment, *Clec16a*^loxP/loxP^ and *UBC-Cre-ER*^T2^-*Clec16a*^loxP/loxP^ were indistinguishable. Approximately 8 days after tamoxifen induction, *UBC-Cre-ER*^*T2*^*-Clec16a*^*loxP/loxP*^ (*Clec16a*^ΔUBC^) mice displayed weight loss that advanced quickly over days (Pandey et al., submitted). Tremors and other mild neurological behaviors (disability score = 1; Fig. 1B) started ~ 10 days and progressed rapidly, with about 60% of mice exhibiting the highest level of disability with dystonic postures after 25 days (disability score = 4; Fig. 1A; see also Supplemental Figure 1). A subset of the mice did not progress beyond a mild or moderate disability even at 40 days, when the experiment was terminated. These mice had higher Clec16a mRNA levels likely due to inefficient LoxP recombination. As observed in a previous study on *Clec16a* mouse models (Redman et al.), *Clec16a*^ΔUBC^ mice exhibited aberrant clasping and/or extension of the hindlimbs instead of the normal splay when picked up by their tails (Supplemental Figure 2). Some *Clec16a*^ΔUBC^ mice exhibited abnormal hind limb posturing by day 11, and all treated mice exhibited this behavior after 30 days. CLEC16A expression was decreased in multiple regions of the nervous system, including those with and without axonopathy (Supplemental Figure 3).

### Diminished amplitude of the caudal nerve action potential in *Clec16a*^ΔUBC^ mice

**Figure 2:**
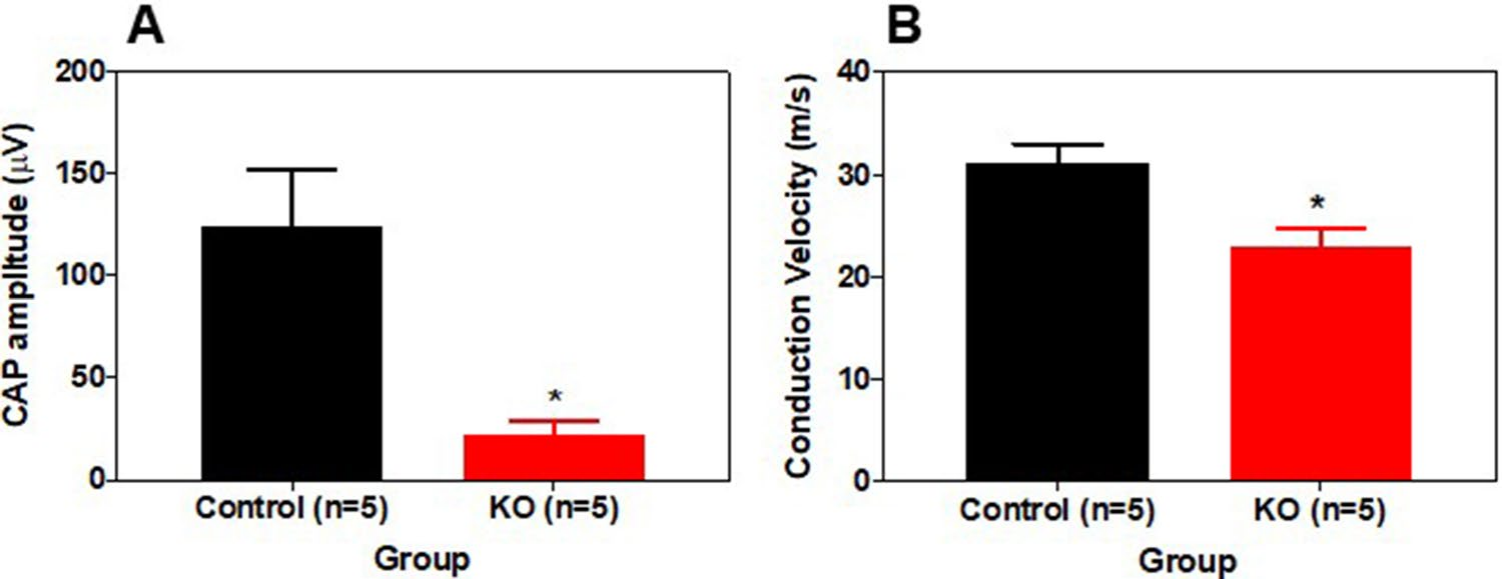
Reduced caudal nerve amplitude and conduction velocity in *Clec16a*^ΔUBC^ mice. The graphs represent mean ± SEM of (**A**) compound action potential (CAP) and (**B**) conduction velocity in the caudal/tail nerves of a cohort of KO/*Clec16a*^ΔUBC^ mice that reached a disability scale of 4, 25 days after initiating tamoxifen treatment, and a cohort of control/*Clec16a*^loxP/loxP^ mice. Number of mice/group indicated on each graph; both sexes were used. *P < 0.05, Student’s *t* test.

The movement disorders we observed in *Clec16a*^ΔUBC^ mice were reminiscent of *dystonic* mutant mice, which have a recessive disorder that result in the progressive degeneration of primary sensory axons^24^. To investigate this issue, we examined *Clec16a*^ΔUBC^ mice that had reached a disability score of 4, 24 days after initiating tamoxifen treatment, along with age-matched control/*Clec16a*^loxP/loxP^ mice. We measured the compound action potentials (CAPs) of the tail, which is a mixed nerve response from the bilateral dorsal and ventral caudal nerves, mainly comprised of myelinated and unmyelinated sensory axons^25–27^. Compared to control/*Clec16a*^loxP/loxP^ mice, *Clec16a*^ΔUBC^ mice had a profoundly smaller CAP amplitude and a mildly decreased conduction velocity (Fig. 2).

### Pathological changes in *Clec16a*^ΔUBC^ mice

**Figure 3:**
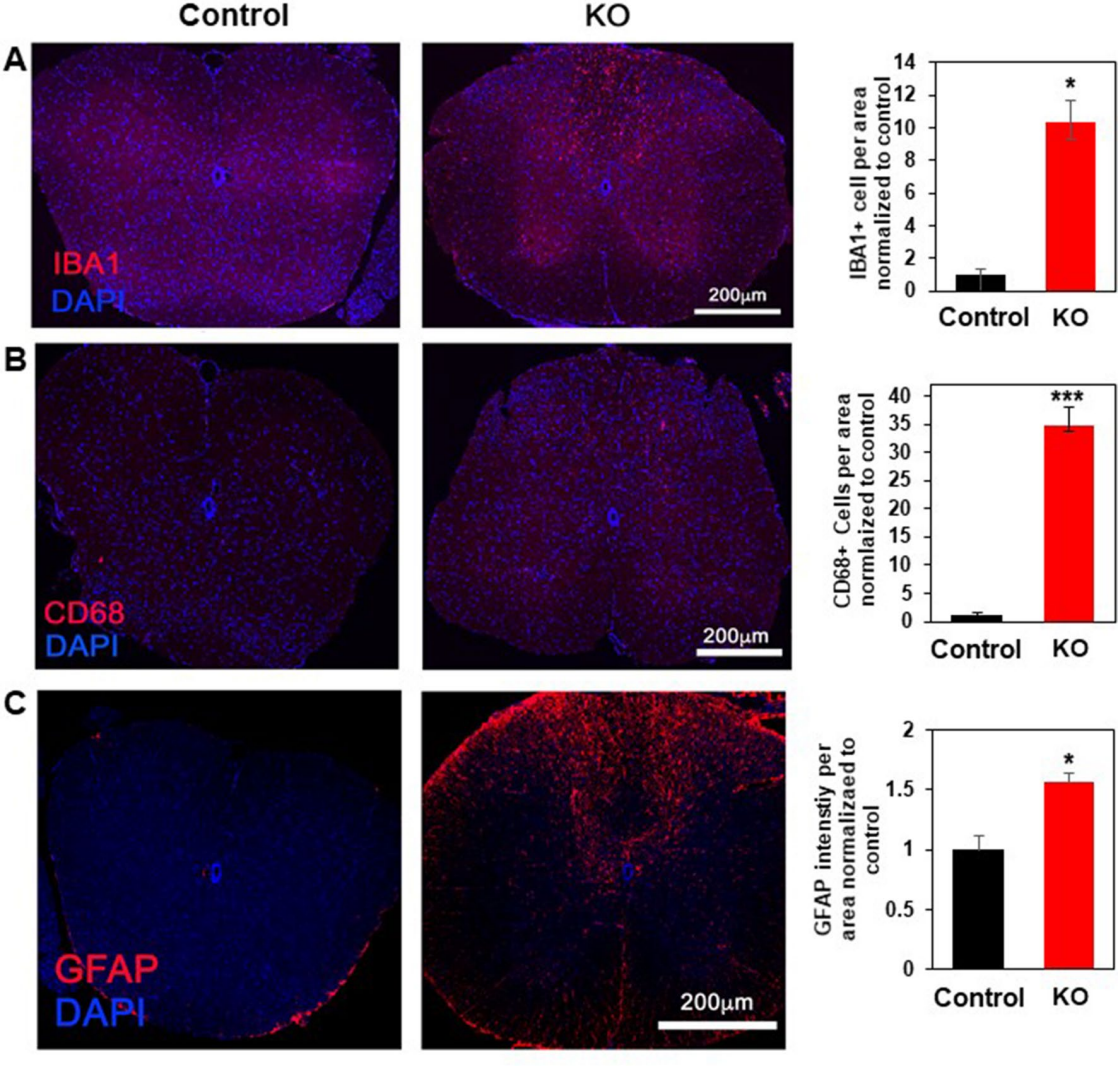
Activated microglia and astrogliosis in the spinal cords, particularly dorsal columns, of *Clec16a*^ΔUBC^ mice. (**A**–**C**) Immunofluorescence images of the spinal cords from control/*Clec16a*^loxP/loxP^ mice and KO/*Clec16a*^ΔUBC^ mice with severe disability and quantification of positive cell labelling. (**A**) Spinal cord sections were labeled with antibody to IBA1 (red), which labels microglia. (**B**) Spinal cord sections were labeled with antibody to CD68 (red) which labels activated microglia. (**C**) Spinal cord sections were labeled with antibody to GFAP (red), which labels astrocytes. For all, the number of positive labeled cells per spinal cord section was counted and divided by the area of the section in three sections from three mice per category and normalized to control. DAPI (blue) labels nuclei. Scale bars = 200 μm. *P < 0.05, ***P < 0.001, Student’s *t* test.

To determine the anatomical location and substrates of the above abnormalities, we first examined various tissues, including the brains and spinal cords, of *Clec16a*^ΔUBC^ mice with the most severe disability vs. control mice, with hematoxylin and eosin (H&E) staining of paraffin sections. These experiments did not reveal any gross morphological changes (data not shown). We next stained frozen sections of the brain and spinal cord with fluoromyelin which labels myelin lipids and ASPA which labels oligodendrocyte cell bodies^28–31^, Calbindin to identify cerebellar Purkinje cells^32^ and GFAP-immunostaining which labels activated astrocytes^33^. This analysis revealed significant overall cerebellar Purkinje cell loss (22%, P = 0.01) and reactive astrocytosis (Supplemental Figure 4), in keeping with a previous report^1^. However, when the number of Calbindin + Purkinje cells and astrocytes were quantified as cells per unit area of cerebellum, a significant difference from controls was not detected (P = 0.17). The average area of the cerebellum was smaller in the *Clec16a*^ΔUBC^ mice, though not significantly (18% smaller, P = 0.12). We found significant increases in CD68-positive cells, IBA1 positive cells, and GFAP positive cells in the dorsal columns (Fig. 3) but not in other brain regions (data not shown). No differences were found between the *Clec16a*^ΔUBC^ mice and control mice in the amount of myelin lipids or oligodendrocyte cell bodies in spinal cord (Supplemental Figure 5).

**Figure 4:**
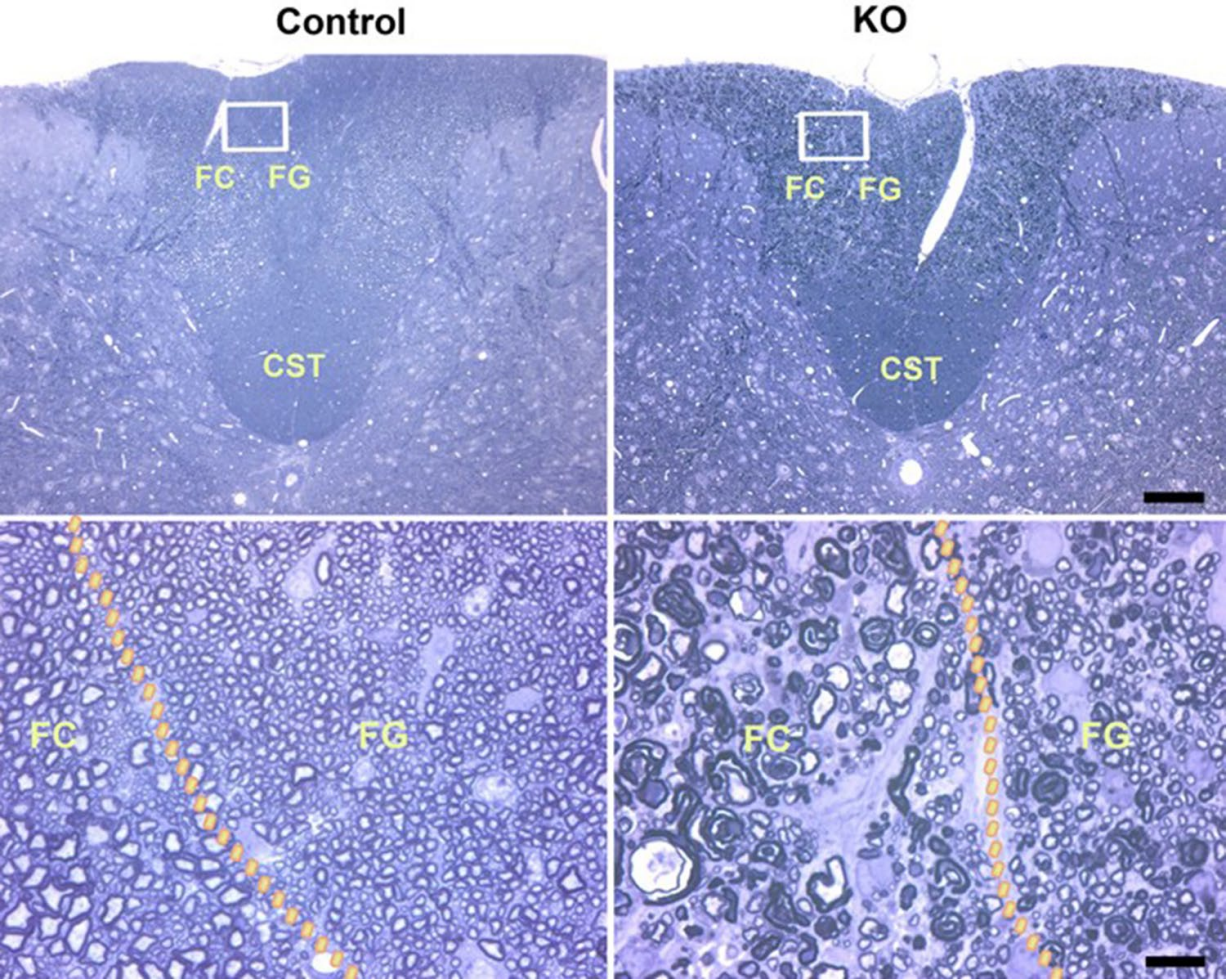
Degenerating sensory axons in the dorsal columns of *Clec16a*^ΔUBC^ mice. These are representative images of semi-thin sections of the lumbar spinal cord from control/*Clec16a*^loxP/loxP^ and KO/*Clec16a*^ΔUBC^ mice with severe disability (score 4), as indicated. The upper panels show the locations of the corticospinal tract (CST), fasciculus gracilis (FG), and fasciculus cuneatus (FC). The lower panels correspond to the rectangular regions depicted in the upper panels, and show many degenerating myelin sheaths in the FG and FG (the boundaries of which are shown by the dotted curves) in the KO/*Clec16a*^ΔUBC^ sample. Scale bar = 100 μm upper panel, 10 μm lower panel.

**Figure 5:**
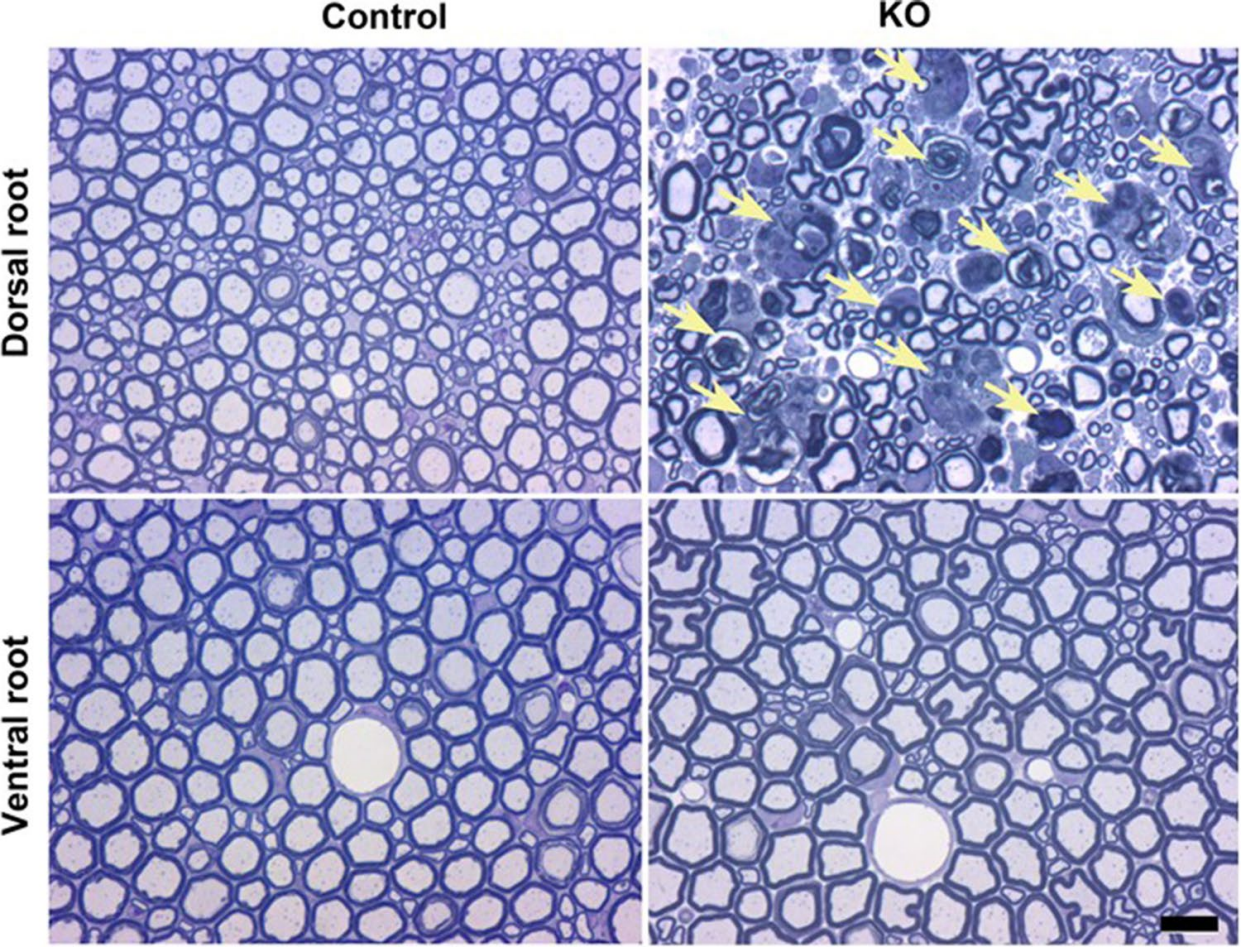
Selective degeneration of sensory and not motor axons in *Clec16a*^ΔUBC^ mice. These are images of semi-thin sections from the dorsal and ventral roots from control/*Clec16a*^loxP/loxP^ and KO/*Clec16a*^ΔUBC^ with severe disability (score 4) mice, as indicated. Degenerating myelinated axons in nerves are only found in the dorsal roots from KO/*Clec16a*^ΔUBC^ mice. Scale bar = 10 microns.

We also processed the spinal cord and various portions of the peripheral nervous system for transmission electron microscopy. In *Clec16a*^ΔUBC^ mice, we found prominent degenerating myelinated axons in the dorsal columns of the lumbar spinal cord, both in the fasciculis gracilis and the fasciculis cuneatus (Fig. 4), which contain the central axons of mechanoreceptors and proprioceptors, respectively^34^, but not in the lateral and ventral funiculi of the spinal cord, which contain ascending and descending axons from diverse types of neurons. In accord, we found degenerating myelinated axons in the dorsal roots, which are comprised only of sensory axons, but not in the ventral roots, which are comprised only of motor axons (Fig. 5). Similarly, we found degenerating myelinated axons in the femoral sensory and motor branches (Supplemental Figure 6) and in the tibial, peroneal, and sural branches of the sciatic nerve (data not shown). Axon size distributions were examined and the frequency of smaller axons in *Clec16a*^ΔUBC^ mice was higher than in control mice (median 5.4 vs 7.4 μm^2^, mean ± SEM. 5.4 ± 0.10 vs 9.7 ± 0.10 μm^2^, respectively; Supplemental Figure 7). This pattern of axonal degeneration, involving dorsal roots in addition to their more distal aspects, is distinct from what one sees in other animal models of peripheral neuropathy, in which one finds distal axonal degeneration, sparing the more proximal axons in the dorsal roots^35^. Because we did not find degenerating sensory neurons in semi-thin sections of the dorsal root ganglia (DRG), it appears that *Clec16a*^ΔUBC^ mice develop a sensory neuropathy that affects proximal and distal axons of both the roots and the peripheral nerves.

To compare the myelin thickness between control and *Clec16a*^ΔUBC^ mice, the ratio between the inner axon radius and the outer myelinated axon radius (g ratio) of femoral sensory nerves from 6 mice (each group) was measured (Supplemental Figure 8). Though a statistically significant decrease was found, the difference between the groups was very small and unlikely to be of biological relevance (control 0.73 ± 0.0008 SEM, n = 5359 neurons, KO group 0.70 ± 0.0011, n = 4326 neurons).

**Figure 6:**
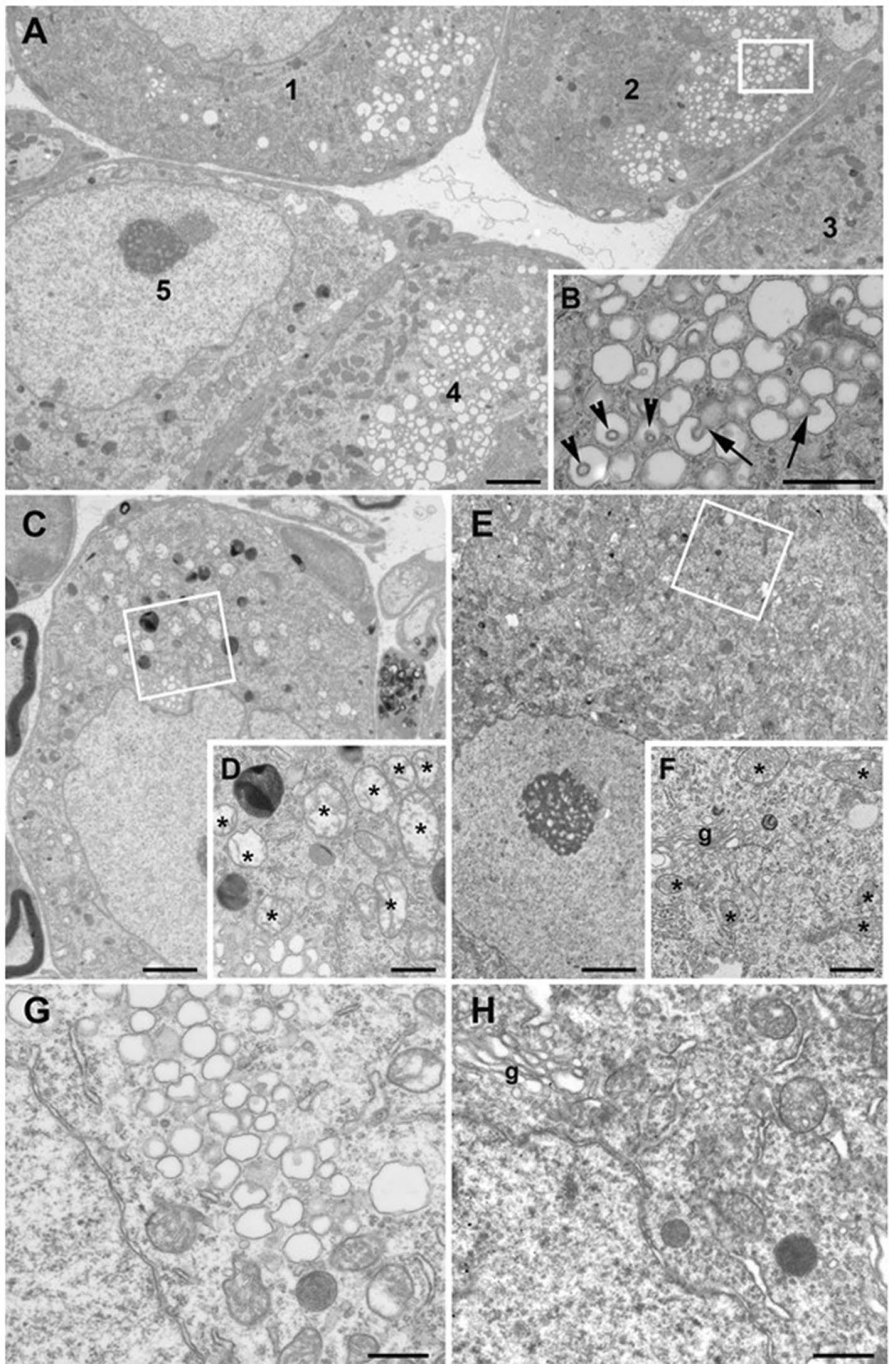
Abnormal vacuoles and mitochondria in *Clec16a*^ΔUBC^ neurons. These are electron micrographs of sensory (A-F) and motor (G&H) neurons from KO/*Clec16a*^ΔUBC^ with severe disability (score 4; **A**–**D**, **G**) and control/*Clec16a*^loxP/loxP^ (**E**,**F**,**H**) mice. In panel (**A**), there are five sensory neurons, three of which (1, 2, and 4) contain cytoplasmic clusters of clear vesicles; the area enclosed by the rectangle is enlarged in panel (**B**). The arrows in panel (**B**) mark two examples of a vesicle that protrudes into an adjacent vesicle; the arrowheads mark three examples in which the origin of the protrusion is not evident in the plane of section. Panel (**C**) shows another sensory neuron that contains abnormal mitochondria labeled in panel (**D**) (asterisks), which is an enlargement of the area shown in the rectangle. Panel (**E**) shows a sensory neuron from a control/*Clec16a*^loxP/loxP^ mouse; the area enclosed by the rectangle is enlarged in panel (**F**), and shows a Golgi apparatus (g) and normal appearing mitochondria (asterisks). Panels (**G**) and (**H**) show portions of cervical motor neurons from a KO/*Clec16a*^ΔUBC^ and a control/*Clec16a*^loxP/loxP^ mouse, respectively. Note the clusters of clear vesicles in the KO/*Clec16a*^ΔUBC^ motor neuron, and the normal appearing Golgi apparatus (g) in the control/*Clec16a*^loxP/loxP^ neuron. Scale bars, 2 μm (**A**,**C**,**E**) and 600 nm (**B**,**D**,**H**).

Clusters of vacuoles were the most conspicuous finding in the DRG neurons of *Clec16a*^ΔUBC^ mice, as previously noted in *Clec16a*-null (*Clec16a*^GT/GT^) cerebellar Purkinje cells^1^ (Fig. 6). In a single, semi-thin section, 11% (range 4–22%) of DRG neurons had clusters of vacuoles (N = 9 animals) in *Clec16a*^ΔUBC^ mice; none were found in *Clec16a*^loxP/loxP^ control mice (N = 5 animals). By electron microscopy (EM; Fig. 6A,B,G), these vacuoles had a single membrane structure, and adjacent vacuoles were seen to protrude into each other, like a finger poking into a balloon, such that some vacuoles appeared to contain a double-membrane structure. We did find clusters of vesicles in a few of the stem processes of DRG neurons (data not shown), but not in the axons of the dorsal and ventral roots or in femoral and sciatic nerves. The vacuoles were postulated to be derived from the Golgi apparatus^1^ but we did not recognize structures that looked like transitional structures between Golgi apparatus and vacuoles, and axons are not known to contain Golgi apparatus.

We also noted vacuoles in a few (> 1%) of the larger neurons in transverse, semi-thin sections of the cervical and lumbar spinal cords of *Clec16a*^ΔUBC^ mice, between 3–13 such neurons were found per section (N = 7 animals), in both the dorsal and ventral horns. None were found in *Clec16a*^loxP/loxP^ control mice (N = 7 animals). By EM (Supplemental Figure 9), a minority of affected neurons had vacuoles that were similar in appearance to those seen in the DRG neurons. Most of the affected neurons, however, had vacuoles that were much larger (Supplemental Figure 9B) than those seen in the DRG neurons, and these affected neurons also had the more typical, smaller vacuoles, too. The relationship between the smaller and larger vacuoles warrants further investigations.

We also noted some DRG neurons (less than 10%) had large numbers of abnormal mitochondria, with an electron-lucent matrix and reduced cristae (Fig. 6C,D). We do not think that these abnormal mitochondria were merely the result of poor fixation, as cells with abnormal mitochondria were found adjacent to cells with normal mitochondria in *Clec16a*^ΔUBC^ mice, and cells with abnormal mitochondria were not found in control mice. In *Clec16a*^ΔUBC^ mice, the cells with abnormal mitochondria usually did not contain clusters of vacuoles.

DRG sensory neurons from *Clec16a*^ΔUBC^ mice also exhibited myelin debris and altered myelin-like structures (Fig. 4, lower right panel). The myelin debris found in the peripheral nerves is likely a consequence of Wallerian degeneration, which occurs when the part of the axon distal to the injury degenerates, followed by degeneration of the myelin sheath and infiltration of macrophages which then clear the debris, as shown previously^36^.

### Alterations in mitophagy-related proteins, endoplasmic reticulum (ER) stress, and oxidative phosphorylation (OXPHOS) related proteins in *Clec16a*^ΔUBC^ mice

Previous reports from our laboratory and others have shown an important role of CLEC16A in mitophagy functions in immune cells, islet β-cells, and cerebellum^1,5–8^. Similarly, in this study we found increased expression of the mitophagy proteins, PINK1, Parkin, and p62, as well as the autophagic protein, lipidated form of LC3 (Supplemental Figure 10). The global effects of a whole-body *Clec16a* deletion on these mitophagy and autophagy proteins contrasts to its specific effects on sensory neurons.

We next examined the effect of *Clec16a* knockout on ER stress and OXPHOS signaling in mitochondrial in light of previous observations^7,37^. Unfolded-protein response and OXPHOS signaling related genes and proteins were examined, focusing on the sensory neuron tissues. The ER stress marker genes were evaluated at mRNA level in sensory neuron tissues, the trigeminal ganglia and DRG, with mild disability (Day = 10) and severe disability (Day = 22). As found previously in other tissues, ER stress markers (GRP78, ATF4, and CHOP) showed significant upregulation at mRNA levels in the *Clec16a*^ΔUBC^ mice with mild and severe disability in both tissues (Supplemental Figure 11A–D). XBP1 was increased in DRG and trigeminal ganglia (TG) of *Clec16a*^ΔUBC^ mice with severe disability. Immunoblot analysis revealed significant increase in expression of CHOP in both DRG and TG’s of mice with severe disability (Supplemental Figure 11C).

We also measured OXPHOS respiratory complex protein levels in sensory neuron tissues (Supplemental Figure 11E–F). The Complex 1 protein, NADH dehydrogenase (ubiquinone) 1 beta subcomplex 8 (NDUFB8) and Complex IV protein, cytochrome c oxidase subunit 2 (COXII), were increased in both DRG and TG of the *Clec16a*^ΔUBC^ mice. The TG of *Clec16a*^ΔUBC^ mice showed an increase of Complex III ubiquinol-cytochrome c reductase core protein II (UQCR2). Complex V protein ATP synthase 5A (ATP 5A) was decreased in both DRG and TG of *Clec16a*^ΔUBC^ mice.

### CLEC16A expression is inversely related to IGS15 protein expression

**Figure 7:**
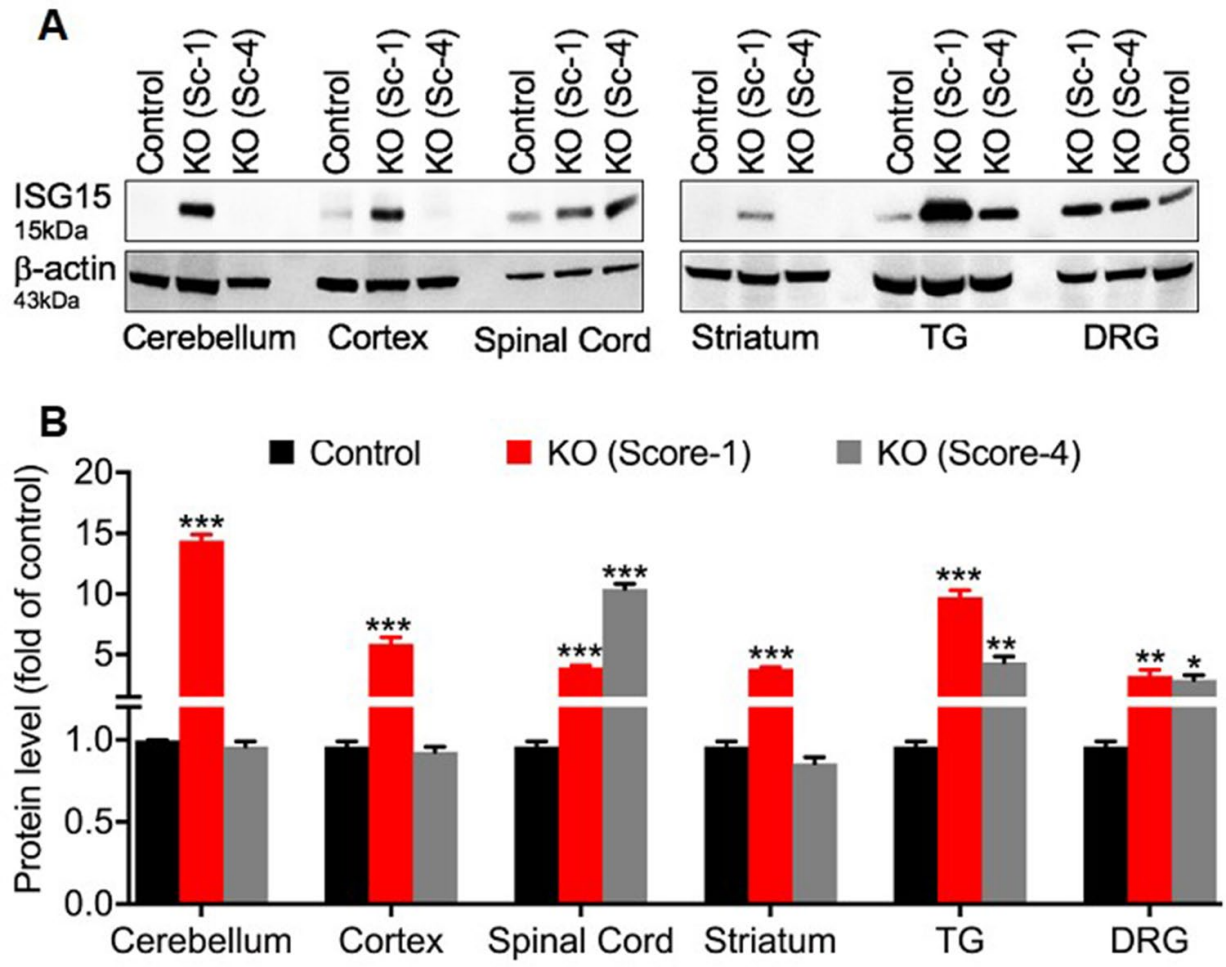
ISG15 expression in neuronal tissues of *Clec16a*^loxP/loxP^ and *Clec16a*^ΔUBC^ mice. (**A**) Representative western blot from cerebellum, cortex, spinal cord, striatum, TG and DRG lysates of control/*Clec16a*^loxP/loxP^ and KO/*Clec16a*^ΔUBC^ mice depicting expression levels of ISG15. Membranes were striped and re-probed for β-actin as a loading control. Image is cropped from the full membrane image; the full membrane image can be found in the Supplemental Figure 12. (**B**) Quantitation graph depicting expression levels of ISG15. Data expressed as mean ± SE of three independent repeats of two mice each. *P < 0.05, ***P < 0.001 (Control vs. KO-Score 1), ^##^P < 0.01 (Control vs. KO-Score 4), Student’s *t* test.

In search of potential candidates interacting with CLEC16A, MSMS analysis was performed on lysates of YTS and YTS-*Clec16a* overexpressing cells immunoprecipitated with CLEC16A. Tripartite motif 25 (TRIM25, aka estrogen-responsive finger protein (EFP)), an E3 ubiquitin, interferon stimulated gene 15 (ISG15) ligase, was identified as one of the top ten hits among the potential candidate proteins interacting with CLEC16A (Supplemental Table 1). A recent study shows TRIM25 is significantly induced upon ER stress, promoting ER-degradation in an attempt to restore ER homeostasis^38^. Because TRIM25 regulates ISG15, ISG15 expression is low under normal conditions, the ubiquitin pathway regulates mitophagy, ISG15 inhibits the ubiquitin pathway, and activation of ISG15 occurs when neuronal damage is present, we hypothesized that a constitutively elevated ISG15 pathway inhibits the ubiquitin pathway and consequently ubiquitin-dependent-mitophagy, which may contribute to neurodegeneration in *Clec16a*^ΔUBC^ mice and CLEC16A’s actions in sensory neurons. We measured ISG15 protein levels in several neuronal tissues in *Clec16a*^ΔUBC^ mice with mild disability (score = 1) and severe disability (score = 4) compared to *Clec16a*^loxP/loxP^/control mice. ISG15 levels in control mice were low as predicted. Significantly increased levels of ISG15 were observed in all neuronal tissues tested from *Clec16a*^ΔUBC^ mice with mild disability compared to controls (Fig. 7). The increase in ISG15 levels was more pronounced in the spinal cord, TG and DRG in *Clec16a*^ΔUBC^ mice with severe disability, while levels were unaltered in cerebellum, cortex or striatum.

## Discussion

We show for the first time that whole body deletion of *Clec16a* in adult mice results in an overt neurological phenotype that is associated with loss of primary sensory axons but not the loss of Purkinje cells seen in other *Clec16a* mutant models. These results are similar to the recent finding that homozygous mice carrying a gene-trap insertion in *Clec16a* (*Clec16a*^GT/GT^) develop an overt neurological phenotype at 7–8 weeks of age, ascribed to the loss of cerebellar Purkinje cells. *Clec16a*^curt/curt^ mice, which have 4-nucleotide deletion allele in exon 21, resulting in a frameshift mutation, also lose cerebellar Purkinje cells^1,39^. It remains to be determined whether primary sensory neurons are affected in *Clec16a*^GT/GT^ and *Clec16a*^curt/curt^ mice. Further studies will be completed to understand why a greater loss of Purkinje cells was not observed in the *Clec16a*^ΔUBC/ΔUBC^ mice.

*Clec16a*^ΔUBC/ΔUBC^ homozygote mice have a severe phenotype and are embryonic lethal based on unequal genotype distribution in heterozygous breeding crosses. In contrast, *Clec16a*^GT/GT^ mice have a delayed and mild behavioral phenotype as well as longer survival^1^; the *Clec16a*^curt/curt^ mice, have decreased body weight, skeletal abnormalities, and prenatal/perinatal death^1,39,40^. In view of the above, we infer that the *Clec16a*^GT^ allele likely produces a partial loss of function.

We observed weight loss and lipodystrophy in the *Clec16a*^ΔUBC^ mice prior to the manifestation of the neuronal phenotype (Pandey et al., submitted). Lipid molecules are important for key brain structure and functions and comprise about 50% of the brain composition; most of this is contained in the myelin sheath around neurons^41^. The neuronal phenotype was not the result of demyelination as myelin-related proteins were not altered in the *Clec16a*^ΔUBC^ mice with severe disability as compared to *Clec16a*^loxP/loxP^/control mice. Likewise, only the sensory neurons were affected and the motor neurons and other CNS structures were intact indicating a global demyelination did not occur. However, ER stress mRNAs were increased in trigeminal and dorsal root ganglia. The brain generates its own phospholipids and, therefore, may be protected from the lipolytic cascade present in adipose tissue. Previous studies show that high stress in neurons stimulates the formation of lipid droplets in nearby glial cells preventing fatty acid toxicity in the neurons themselves serving as neuronal protection^42^.

Sensory neurons are responsible for conveying external environmental stimuli to the central nervous system. When there is a loss of these neurons, the transmission of information is incomplete and consequently the brain cannot send motor impulses to respond correctly to its surroundings. The selective involvement of cerebellar and/or primary sensory neurons in *Clec16a* KO mice models a human syndrome known as spinocerebellar ataxia, which has diverse systemic and genetic causes^43–45^. Though CLEC16A has not been implicated in human spinocerebellar ataxia, other mitophagic and autophagic proteins have been identified. Some mouse models of other autophagocytic-related proteins exhibit gait abnormalities, ataxia, cerebellar/Purkinje cell deficits, fewer dendritic spines, axonal swelling, and other CNS abnormalities. There are few mouse models of a sensory neuronopathy—*Atg7* conditional KO using Advillin-Cre mice, and *Dst*^*dt*^ transgenic mice^46,47^. Our *Clec16a*^ΔUBC^ mice model represents a sensory ataxia with dystonia, immune and inflammatory components seen in some human cerebellar ataxias and sensory neuronopathies^45,48^.

Sensory neuropathy and/or cerebellar degeneration have been observed in autoimmune disorders that are associated with *CLEC16A* such as type I diabetes, celiac disease, multiple sclerosis, systemic lupus erythematosus, autoimmune thyroid disease, and systemic sclerosis (scleroderma)^45,49–55^. The connection between *CLEC16A* variants and sensory neuropathy or cerebellar degeneration have not been considered clinically in any of these disorders. Cerebellar Purkinje cells express high levels of CLEC16A^23^ which may contribute to some loss of cells in the *Clec16a*^ΔUBC^ mice. Others have noted that mechanisms underlying spinocerebellar ataxia, as well as dystonia, involves specific pathways likely crucial for Purkinje cell function^43,56^, indicating CLEC16A is connected to one of these pathways.

The role of TRIM25 has been further developed recently to not only include its role in innate immunity^57,58^ and metabolism^59^ but also to oncology^38,60^ and mediating clearance of misfolded proteins^61^. It is unclear if TRIM25 is interacting with CLEC16A and affecting neuropathy in our *Clec16a*^ΔUBC^ mice. Future studies will address the direct CLEC16A interaction with TRIM25 and its function or impact in neuronal loss. The contribution of IGS15 to neurodegeneration by inhibiting the ubiquitin pathway is intriguing, but not yet confirmed^19^. One role of free ISG15 is stimulating the production of IFN-γ from CD3^+^ T cells to enhance the proliferation and cytotoxicity of natural killer (NK) cells^22^. A recent study found that stimulated NK cells degenerate partially injured sensory axons^62^. Since increased NK cell cytotoxicity is observed in the *Clec16a*^ΔUBC^ mice^37^, future studies could address whether this is a potential mechanism for selective sensory neuron loss.

Autophagy and apoptosis are important and interconnected stress response mechanisms. Dead or damaged cells constitute a source of novel antigens and proinflammatory molecules that can provoke autoimmune inflammatory immune response. ISG15 is in the type-I-interferon (IFN) signaling cascade and is increased after cell stress, in neuronal injury and motor neuron disease (ALS) in humans and mice^15,16,63,64^. A role for ISG15 in the mitochondria is established as 17% of free ISG15 and about 5% of total ISG15 target proteins are localized in the mitochondria. ISG15 and ISGylation are implicated in the control of mitochondrial OXPHOS and recycling in bone marrow-derived macrophages^20^. We found increases in ISG15 in neuronal tissues during the early phases of neuronal disability with a concomitant decrease in the brain regions at the later, more severe disability timepoint. Upregulation of ISG15 has been noted in several neuronal injury models involving inflammation and in a mouse model of ALS motor neuropathy^16,64^. However, in the models described, ISG15 was not increased in unaffected CNS areas. These data suggest that CLEC16A directly or indirectly more broadly impacts IGS15 activation and protein ISGylation, which are associated with enhanced and prolonged JAK-STAT signaling, initiating SRC family tyrosine kinases, and ultimately increased cytokine and chemokine production in the *Clec16a*^ΔUBC^ mice.

Targeting any of these pathways often induces harmful side effects on top of positive effects thereby negating potential benefits. While our current exploratory intervention experiments in the *Clec16a*^ΔUBC^ mice show only partial rescue of the phenotype (Pandey et al., submitted), newer, more selective inhibitors targeting IFN-STAT signaling (Ruxolitinib) alone or in combination with autophagy/mitophagy modulators, represent potential opportunities for autoimmune and inflammatory disorders without the harmful side effects^65^.

The complexity of each condition, and the presence of different types of neuropathies across different autoimmune diseases, complicates the elucidation of how CLEC16A is related to sensory neuron loss. Whether the above altered pathways contribute to the selective loss of primary sensory neurons in *Clec16a*^ΔUBC^ mice remains to be determined. Autophagy is necessary for neuronal homeostasis and survival^66,67^, but this alone would not account for the selective vulnerability of affected neurons. Nor would astrogliosis and activated microglia be the logical explanation, as both are found in a wide variety of CNS insults. The *Clec16a*^loxP/loxP^ mice provide a valuable murine model to elucidate these issues in sensory ataxia as well as mitochondrial function in neurodegeneration.

## Materials and methods

The study is reported in accordance with ARRIVE guidelines.

### Mice

The Institutional Animal Care and Use Committee of the Children’s Hospital of Philadelphia approved all animal studies. All methods were performed in accordance with the IACUC guidelines and regulations. Mice were group-housed on an individually-ventilated cage rack system on a 12:12 light:dark cycle. Mice were fed standard rodent chow and water ad libitum.

Mice were generated as described previously^8^ *Clec16a*^loxP^ mice were generated by flanking exon 3 (Ozgene). Mice with targeted insertion in the *Clec16a* gene were crossed to the Flpo Deleter line (mouse Strain: 129S4/SvJae-Gt (ROSA) 26Sortm2(FLP*) Sor/J; The Jackson Laboratory) to achieve deletion of the FRT-flanked Neomycin cassette. *Clec16a*^loxP^ mice were mated to UBC-*Cre-ER*^*T2*^ mice (inducible cre recombinase driven by the human ubiquitin C promoter) obtained from The Jackson Laboratory to generate UBC-*Cre*-*Clec16a*^loxP^ mice. Mice were kept on a mixed background C57BL/6-129S1.

Ten-week old *UBC-Cre-ER*^T2^-*Clec16a*^loxP/loxP^ male and female mice were treated orally by gavage with tamoxifen (100 mg/kg/day) at 24-h intervals for four consecutive days to create experimental *Clec16a*^ΔUBC^ groups. *Clec16a*^loxP/loxP^ littermates dosed with either tamoxifen or vehicle (10% ethanol:90% corn oil) were used as controls. There were no differences in behavior between the two control groups, so they were combined for phenotypic analyses. All images depicting mouse behavior were taken by one of the authors of the manuscript.

### Behavioral observations

Mice were an average of 10 weeks old at beginning of study. Mice were weighed 3 times a week. Mice were scored for gross abnormalities and behavior, including disability and hindlimb splay.

Disability was scored 0–4 as follows:

- 0 = Normal behavior, appearance and movement
- 1 = No impairment; abnormal behavior or appearance (ex: tremor, not grooming)
- 2 = Mild impairment; uneven gait (ex: waddling, abdomen pressed to floor)
- 3 = Moderate impairment; body constrictions, unsteady gait upon initiation of movement
- 4 = Severe impairment; abdominal twisting and limb contractions upon initiation of movement, little to no forward movement, ambulation impaired

Hindlimb splay was scored 0–2 as follows:

- 0 = normal, hind limbs are (splayed) out when lifted
- 1 = One hind limb is partially grasping or contracted out towards tail; abnormal, one or both hind limbs are drawn partially into the body or partially grasping more than 50% of observation time
- 2 = Both hind limbs are grasping together immediately; impaired, both hind limbs are grasping together or contracted out towards tail more than 50% of observation time

Observations were completed prior to treatment and then 3 times per week until clinical signs were noticed at which point mice were observed and scored daily. Once a mouse reached the highest disability score of ‘4’ or greater than 20% body weight loss from baseline, it was euthanized and tissues were collected. Some mice were euthanized at different disability scores (without the opportunity to progress a more severe score) to understand the molecular mechanism of CLEC16A. Mice remaining at the end of the study (40 days) were euthanized and organs were harvested for future analyses.

### Nerve conduction study

A portable EMG/NCS machine (Nicolet) was used to record the CAPs from caudal nerves. Mice were anesthetized with ketamine: xylazine (10:100 mg/kg, IP). After full anesthesia was verified by toe pinch, mice were placed supine with the tail fully extended. Electrodes were placed on the tail following the method of Maia et al.^25^ Wire loop electrodes were used for recording and placed with the reference loop 0.2 cm from the tail base and the active electrode 0.5 cm more distal along the tail. One-pin electrodes were used as ground electrodes (separated by 0.6 cm). More distally along the tail, a pair of loop electrodes was used for stimulation (separated by 0.5 cm). The distance between the recording electrode and stimulating cathode electrode is three cm. The intensity of electrical stimulation for mice is equivalent to the intensity used in clinical nerve conduction studies in awake human. A series of 5 responses were averaged to reduce noise and variability. Tail surface temperature was maintained at 30–32 °C using a heating pad and monitored using infrared non-contact thermometer during recording. Upon completion of recording, mice were returned to the cage and closely observed until they fully recovered.

### Western blot

As described in Pandey et al*.*^8,37^, sample lysis was performed with NP40 lysis buffer (Invitrogen). The lysates were electrophoresed on 4–12% NuPAGE Bis–Tris gels in MOPS SDS running buffer and transferred onto nitrocellulose membranes (Invitrogen). The membranes were blocked in 3% BSA and incubated with indicated primary antibodies where specified: CLEC16A, PINK1 (Abgent), Parkin, p62/SQSTM1, LC3 I/II for mitophagy/autophagy (Santa Cruz), CHOP for ER stress (Novus Biologicals) and ISG15 (Cell Signaling Technology). For OXPHOS signaling, a commercially available cocktail antibody (ab110413, Abcam) comprising the following subunits of respiratory complex proteins were used: NADH dehydrogenase (ubiquinone) 1 beta subcomplex 8 (NDUFB8; complex I), succinate dehydrogenase complex, subunit B, iron sulfur (SDHB/Ip; complex II), ubiquinol-cytochrome c reductase core protein II (UQCR2; complex III), cytochrome c oxidase subunit 2 (COXII; complex IV) and ATP synthase 5A (ATP 5A, Complex V).

The membranes were washed and incubated with a respective secondary antibody as recommended by the manufacturer. Bound antibody was detected with WesternBright ECL chemiluminescence detection system (Advansta, San Jose, CA). Membranes were stripped and re-probed with mouse anti-β-Actin mAb (Abcam) as a loading control. Membranes were cut in parts and probed for protein of interest where specified. Band intensities were measured using Image J software (v1.53, NIH Shareware), scanned in grey scale mode at 300 DPI and saved in TIFF format, measuring the area under each peak for each band.

### Quantitative real-time PCR

As described in our previous publication^37^, total RNA was isolated with Trizol reagent (Invitrogen) following RNA purification using the RNeasy Mini Kit (Qiagen) and converted to cDNA by High Capacity RNA-to-cDNA Kit (Applied Biosystems), according to the manufacturer’s protocols. Assay comprising known human and murine *CLEC16A* RNA transcripts as well as control genes (*β-actin* and *HRPT1*) were measured by real time PCR on a ViiA 7 Real Time PCR System using predesigned 20X FAM-MGB TaqMan gene expression assays available from Applied Biosystems. All assays had primers covering exon-exon boarders to avoid DNA contamination. Triplicates were used for all samples included in the experiment. All PCR runs were performed on ViiA 7 Real Time PCR System using ViiA7 RUO software v1.2.2 (Life Technologies).

### Immunohistochemistry

Mice were euthanized, perfused with 4% paraformaldehyde, brains were embedded in OTC, and frozen sections were cut on a Leica cryostat at 12-micron thicknesses, all according to established protocols^68,69^. Hematoxylin and eosin (H&E) staining was performed by the histology core at Children’s Hospital of Philadelphia. Oligodendrocytes were labeled with fluoromyelin which labels myelin lipids and ASPA which labels oligodendrocyte cell bodies^28–31^. Microglia/macrophages were labeled with anti-IBA-1 (Wako Pharmaceuticals, 1:1000) and anti-CD68 (Abcam, 1:100)^70,71^. Astrocytes were labeled with used anti-GFAP antibody (rat monoclonal, undiluted, gift of Dr. Virginia Lee, University of Pennsylvania)^33^. Purkinje cells were labeled with an anti-calbindin antibody (Swant, 1:2000)^32^. Sections were incubated with primary antibodies overnight at 4 °C. Secondary antibodies of appropriate species and isotype used for external and internal antigens were purchased from Jackson Immunoresearch, West Grove, PA and used at 1:200 for 30 min. Coverslips were mounted onto glass slides in 4′,6-diamidino-2-phenylindole (DAPI)-containing Vectashield mounting medium (Vector Laboratories, Burlingame, CA, USA). For all, the number of positive labeled cells per spinal cord or cerebellar section was counted and divided by the area of the section in three sections from three mice per category and normalized to control.

### Transmission electron microscopy

Anesthetized mice were transcardially perfused with 2% paraformaldehyde and 2% glutaraldehyde in 0.1 M phosphate buffer (pH 7.4). DRG and cervical spinal cords were dissected, and fixed for at least 4 more hours at 4 °C. The tissues were osmicated, dehydrated, infiltrated, and then embedded in Embed 812 mixture (Electron Microscopy Sciences) as previously described^72^. For light microscopy, cross-sections were cut at a thickness of 1 μm on an American Optical Reichert Ultracut Ultra microtome, and stained with alkaline toluidine blue. For electron microscopy, cross-sections were cut at a thickness of 90 nm, and stained with lead citrate and uranyl acetate. The ultra-thin sections were imaged using a JEOL 1010 electron microscope.

### Axon size distribution and measurement of g ratio of femoral sensory nerves

Femoral sensory nerves from six mice in each group (KO and control) were analyzed. All the myelinated axons of one LM images (× 100) from each mouse were analyzed. The g-ratio was calculated by the square root of the ratio of inner to outer axonal area since most axons were not perfectly circular. Inner and outer axonal area were measured using Image J software (v1.53, NIH Shareware).

### Mass spectrometry analysis of Clec16a

Methods for the mass spectrometry are as described in Greco et al.^73^ As described in Pandey et al.^37^, the YTS cell line were grown in 5% CO_2_ at 37 °C in complete RPMI medium (Gibco). The “complete” medium indicates it was supplemented with 10% FBS (Thermo Scientific), non-essential amino acids, HEPES, l-glutamine, sodium pyruvate, and penicillin–streptomycin (all from Gibco). The YTS-*CLEC16A* (Clec16a overexpressing) cell line^37^ was grown in 5% CO_2_ at 37 °C in complete RPMI medium as described above with G418 (1.6 g/L, Mediatech). Briefly, 5 × 10^6^ YTS-CLEC16A and YTS cells were lysed in ice cold IP-lysis buffer. After centrifugation at 13,000 rpm for 10 min at 4 °C, supernatants were pre-cleared with 50 μl of agarose G beads (Invitrogen) and IgG for 45 min at 4 °C. The pre-cleared lysates were incubated with CLEC16A antibody for 1 h at 4 °C and then with 50 μl of agarose G beads for an additional 1 h at 4 °C. Immune complexes were washed three times, dissolved in SDS sample buffer, and ~ 50 μg was resolved by SDS-PAGE. Gel regions were excised from the Coomassie stained gels and cut into 1 mm^3^ cubes (2, 3)(2, 3)(2, 3)(2, 3)(2, 3)(2). The latter served as controls for gel loading and internal standards for MS analysis. The excised gel pieces were then washed with 100 μl of 50 mM ammonium bicarbonate, followed by 50 mM ammonium bicarbonate in 50% acetonitrile and then solvent removal in a vacuum centrifuge. Dehydrated gel pieces were then reswollen in 100 μl of a digestion buffer containing 50 mM ammonium bicarbonate, and 12.5 ng/ml of trypsin and enzymatic cleavage was allowed to continue overnight at 37 °C. Peptides were extracted into 20 mM ammonium bicarbonate (100 μl) followed by two separate extractions into 100 μl of water/acetonitrile/formic acid (10:10:1; v/v/v). After evaporation to dryness, peptides were re-dissolved in 10 μl of 5% acetonitrile and 0.1% FA for LC/MS/MS analysis. For peptide mass fingerprinting of samples, 0.5 μl of each Tryptic digest was mixed with 1 μl of matrix (saturated aqueous solution of 2,5-dihydroxybenzoic acid) and spotted onto a sample target plate and dried. Matrix-assisted laser desorption/ionization time-of-flight (MALDI-TOF) mass spectra were acquired using an externally calibrated Ultraflex TOF/TOF mass spectrometer (Bruker Daltonics) in the reflector mode. After internal calibration using trypsin autolysis peaks, prominent peaks in the mass range m/z 700–4000 were used to generate a peptide mass fingerprint that was searched against the updated human IPI database using Mascot version 2.1.3 (Matrix Sciences, London, UK). Identifications were accepted when a minimum of six peptide masses matched to a particular protein (mass error of ± 50 ppm allowing 1 missed cleavage), sequence coverage was > 25%, MOWSE scores were higher than the threshold value (P = 0.05) and the predicted protein mass agreed with the gel-based mass.

Nano-HPLC electrospray ionization (ESI) collision induced dissociation (CID) MS/MS was performed on a Q-TOF mass spectrometer (Waters, Manchester, UK), coupled to an Ultimate system LC (Dionex) with a PepMap C18 75 μm inner diameter column at a flow rate of 300 nl/min. Spectra were processed using MassLynx software (v4.0, Waters) and submitted to Mascot database search routines. Positive identifications were made when at least two peptide sequences matched an entry and MOWSE scores were above the significance threshold value (P = 0.05). The data analysis and sample report were created using Proteome Software Scaffold (version: Scaffold_3.3.3).

### Statistical analysis

All graphs denote mean values, except where noted, and error bars represent the SEM. Comparisons of groups of data were performed using Log-rank (Mantel-Cox) test for survival curves or two-tailed Student’s *t* tests for parametric data (Prism ver7, GraphPad Software, Inc.). Statistical significance is shown in figures (*P < 0.05, **P < 0.01, ***P < 0.001). No phenotypic difference was observed between the *Clec16a*^loxP/loxP^ treated with vehicle or *Clec16a*^loxP/loxP^ mice treated with tamoxifen, so data were combined for comparison to *Clec16a*^ΔUBC^ mice.

## Supplementary Information

Supplementary Table 1.

Supplementary Figures.
