# Supplementary material for: Inducible knockout of Clec16a in mice results in sensory neurodegeneration
Source: Sci Rep. 2021 Apr 29;11:9319. doi: 10.1038/s41598-021-88895-0 (PMC8084945; doi:10.1038/s41598-021-88895-0)
Supplement: Supplementary file 2 — Supplementary Figures. [file 41598_2021_88895_MOESM2_ESM.docx]

**Title: Inducible knockout of *Clec16a* in mice results in sensory neurodegeneration**

**Authors:** Heather S. Hain*^1^, Rahul Pandey^1^, Marina Bakay^1^, Bryan P. Strenkowski^1^, Danielle Harrington^1^, Micah Romer^2^, William W. Motley^2^, Jian Li^2^, Eunjoo Lancaster^2^, Lindsay Roth^2^, Judith B. Grinspan^2,3^, Steven S. Scherer^2,5^, and Hakon Hakonarson*^1,4,5^

**Affiliations:**

^1^The Center for Applied Genomics; Children’s Hospital of Philadelphia; Philadelphia, PA, 19104; USA.

^2^Department of Neurology, the Perelman School of Medicine; University of Pennsylvania; Philadelphia, PA, 19104; USA.

^3^Department of Neurology; Children’s Hospital of Philadelphia; Philadelphia, PA, 19104; USA.

^4^Department of Pediatrics, the Perelman School of Medicine; University of Pennsylvania; Philadelphia, PA, 19104; USA.

^5^Co-senior authors

***Corresponding authors:** Hakon Hakonarson [hakonarson@email.chop.edu](mailto:hakonarson@email.chop.edu), Heather Hain [hainh@email.chop.edu](mailto:hainh@email.chop.edu)

**Supplemental Figure 1.**

**Supplemental Figure 1. *Clec16a*^ΔUBC^ (whole-body, inducible knockout) mice exhibit neurological disability.** Graph illustrating average of disability (scores 1-4) over days in a cohort of control/*Clec16a*^loxP/loxP^ and KO/*Clec16a*^ΔUBC^ mice after treating P73-79 mice with tamoxifen for 4 successive days. Number of mice/ group indicated on the graph; both sexes were used. *P<0.05, ***P<0.001 (Control vs. KO) for each day as analyzed by two-way ANOVA with Bonferroni’s correction for multiple comparisons**.**

**Supplemental Figure 2.**

**
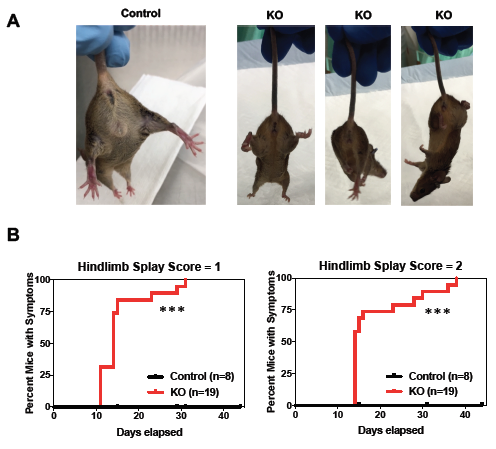
**

**Supplemental Figure 2. *Clec16a***^ΔUBC^ **mice display aberrant hindlimb clasping and extension**. (A) Image demonstrating normal hindlimb splay in control/*Clec16a*^loxP/loxP^ mice and aberrant hindlimb clasping or extension in three different KO/*Clec16a*^ΔUBC^ mice. (B) Timeline and occurrence of abnormal hindlimb clasping (scores 1-2) onset in KO/*Clec16a*^ΔUBC^ mice after initiation of tamoxifen treatment. Number of mice/ group indicated on each graph. ***P<0.001 (control vs. KO) for each score.

**Supplemental Figure 3.**

**
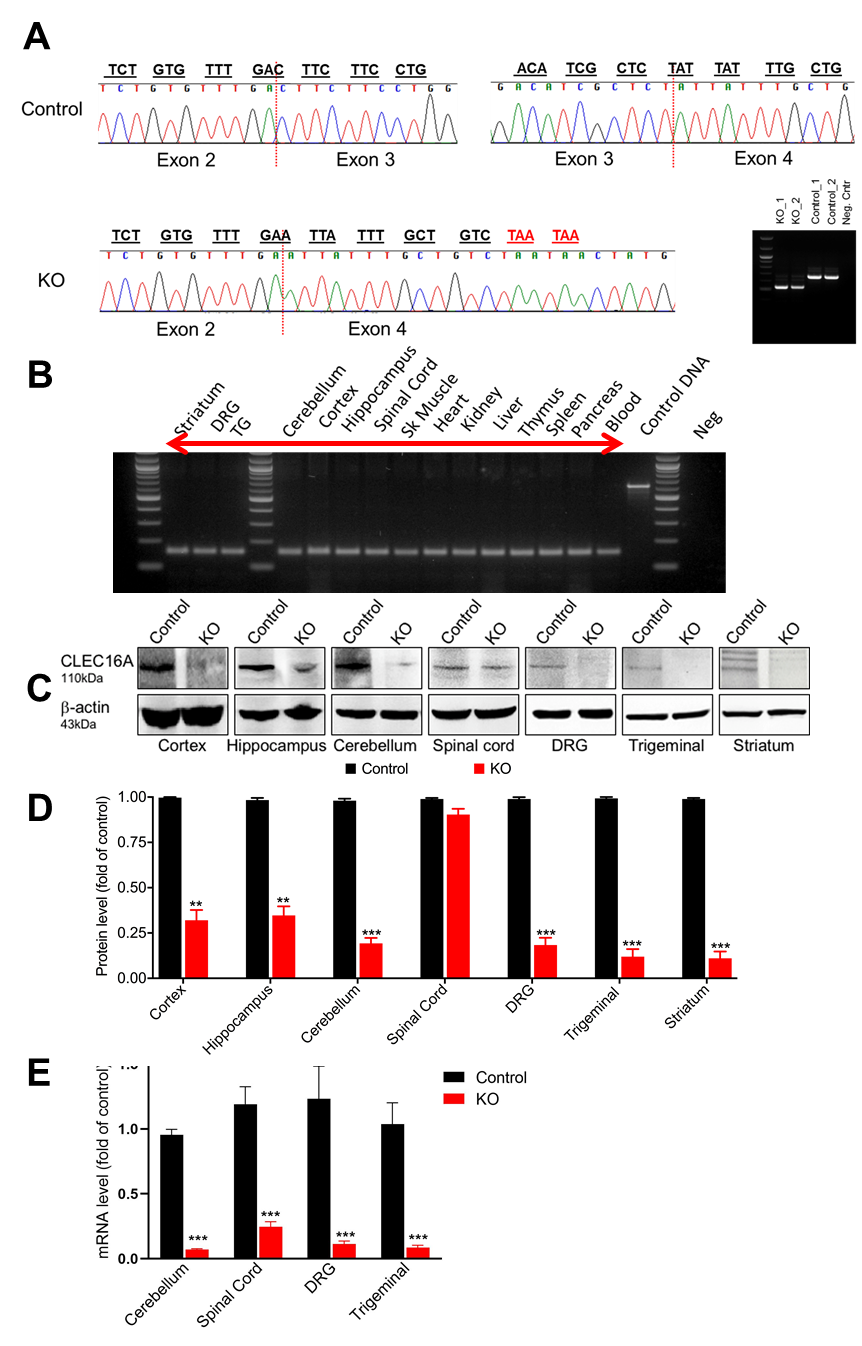
**

**Supplemental Figure 3. CLEC16A expression in control and *Clec16a***^Δ^**^UBC^ mouse tissues.** (A) Direct Sanger sequencing of cDNA from blood, cerebellum and DRG confirmed induced skipping of exon 3, frameshift of the reading frame and appearance of STOP codons in exon 4. Agarose gel electrophoresis shows RT-PCR products produced for KO/*Clec16a*^ΔUBC^ and controls (right) that were used for sequencing. (B) CLEC16A mRNA levels in different control/*Clec16a*^loxP/loxP^ and KO/*Clec16a*^ΔUBC^ mouse tissues as measured by RT-PCR. 18-22 days post tamoxifen treatment. Image is representative of six different KO/*Clec16a*^ΔUBC^ mice. (C) Representative Western blot depicting CLEC16A expression in whole cortex, thalamus, cerebellum, spinal cord, dorsal root ganglia (DRG), trigeminal ganglia, and striatum lysates of control/*Clec16a*^loxP/loxP^ and KO/*Clec16a*^ΔUBC^ mice. β-actin was used as the loading control. (D) Graph depicting reduced CLEC16A protein expression in KO/*Clec16a*^ΔUBC^ neuronal lysates. (E) Graph depicting reduced mRNA in KO/*Clec16a*^ΔUBC^ neuronal lysates as measured by quantitative RT-PCR. Data presented as Mean±SE of three independent repeats. **p<0.01 from control; ***p<0.001 from control (unpaired two-tailed Student’s t-test).

**Supplemental Figure 4.**


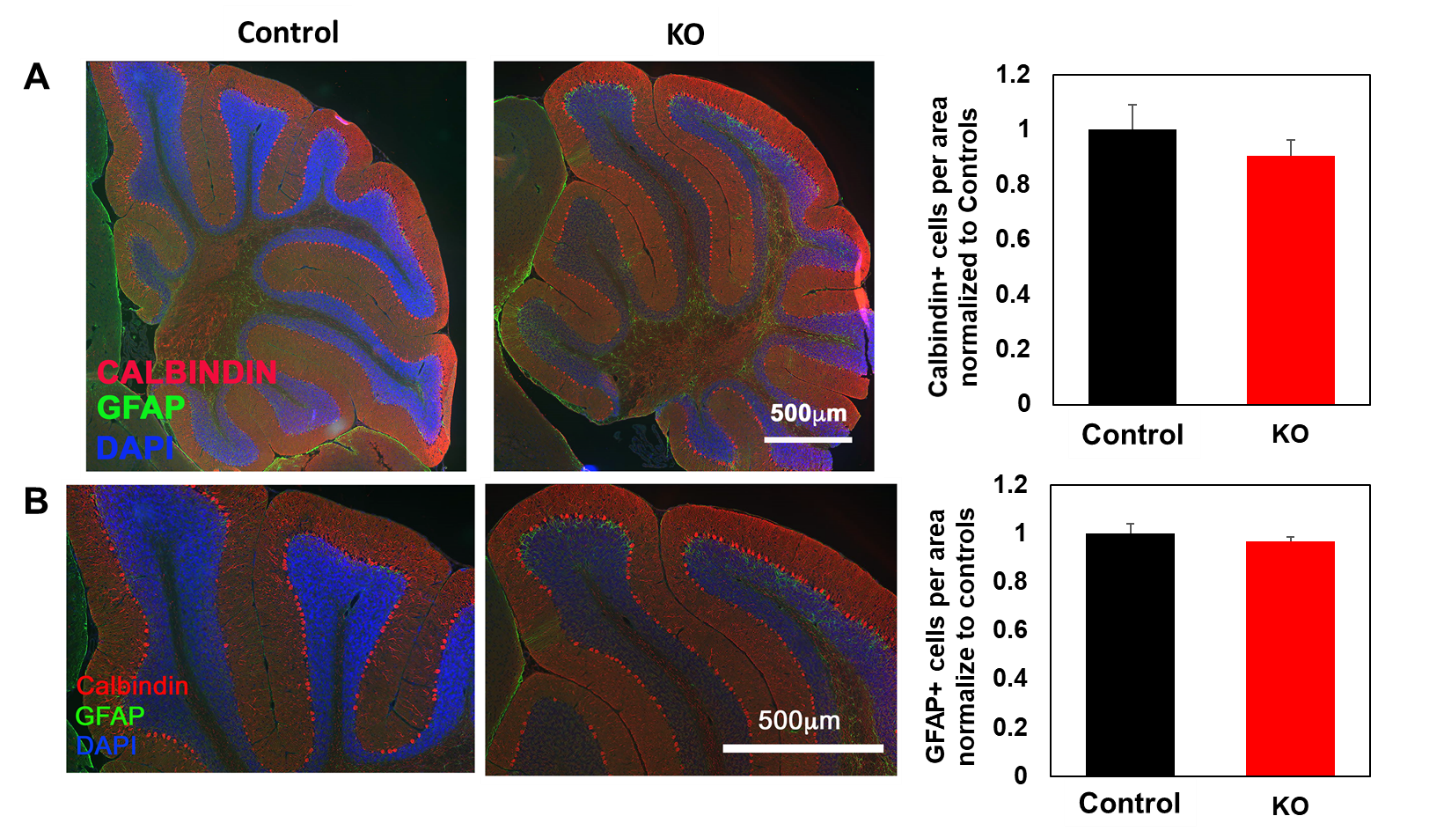


**Supplemental Figure 4. *Clec16a***^ΔUBC^ **mice do not exhibit cerebellar Purkinje cell loss per unit area**. (A-B) Immunofluorescence images of the cerebellum from control/*Clec16a*^loxP/loxP^ mice and KO/*Clec16a*^ΔUBC^ mice with severe disability and quantification of positive cell labelling. (A) Sections of cerebellum were labeled with antibody to calbindin (red) which stains Purkinje cells. (B) Sections from cerebellum were labeled with antibody to GFAP (green) which stains astrocytes. For all, the staining intensity per unit area was quantified in three sections per mouse in three mice per category and then normalized to controls. Scale bars = 500μm. DAPI (blue) labels nuclei.

**Supplemental Figure 5.**

**
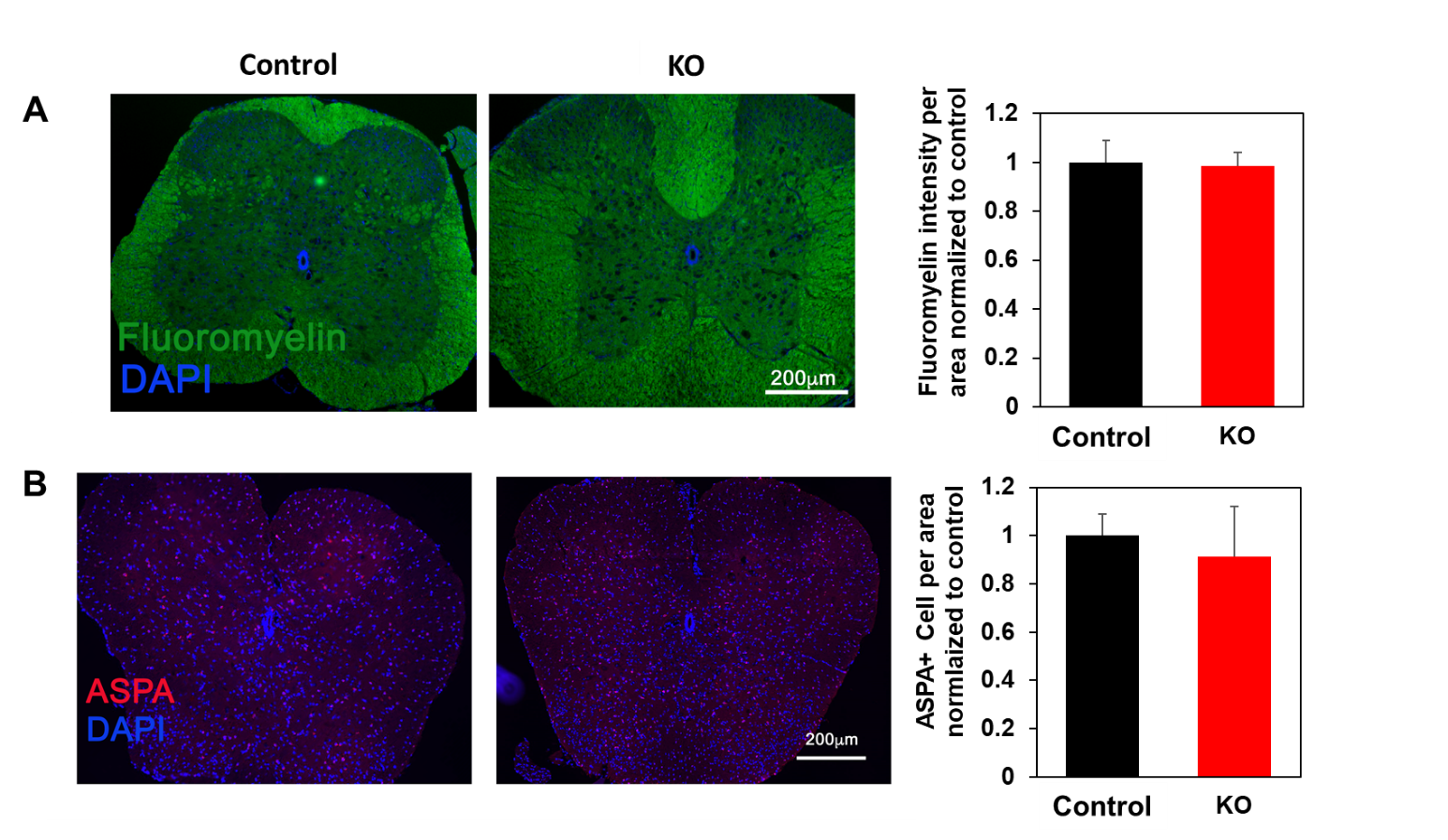
**

**Supplemental Figure 5. No differences in myelin labeling or numbers of oligodendrocytes in spinal cord sections from *Clec16a***^ΔUBC^ **compared to control** **mice.** (A-B) Immunofluorescence images of the spinal cords from control/*Clec16a*^loxP/loxP^ mice and KO/*Clec16a*^ΔUBC^ mice with severe disability and quantification of positive cell labelling. (A) Spinal cord sections were labeled with fluoromyelin which stains myelin lipids and staining intensity per area of spinal cord section was determined in three sections from three mice per category and normalized to control. (B) Spinal cord sections were labeled with antibody to ASPA which labels oligodendrocyte cell bodies and the number of ASPA+ cells per spinal cord section was counted, divided by area, in three sections from three mice per category and normalized to control. DAPI (blue) labels nuclei.

**Supplemental Figure 6.**

**
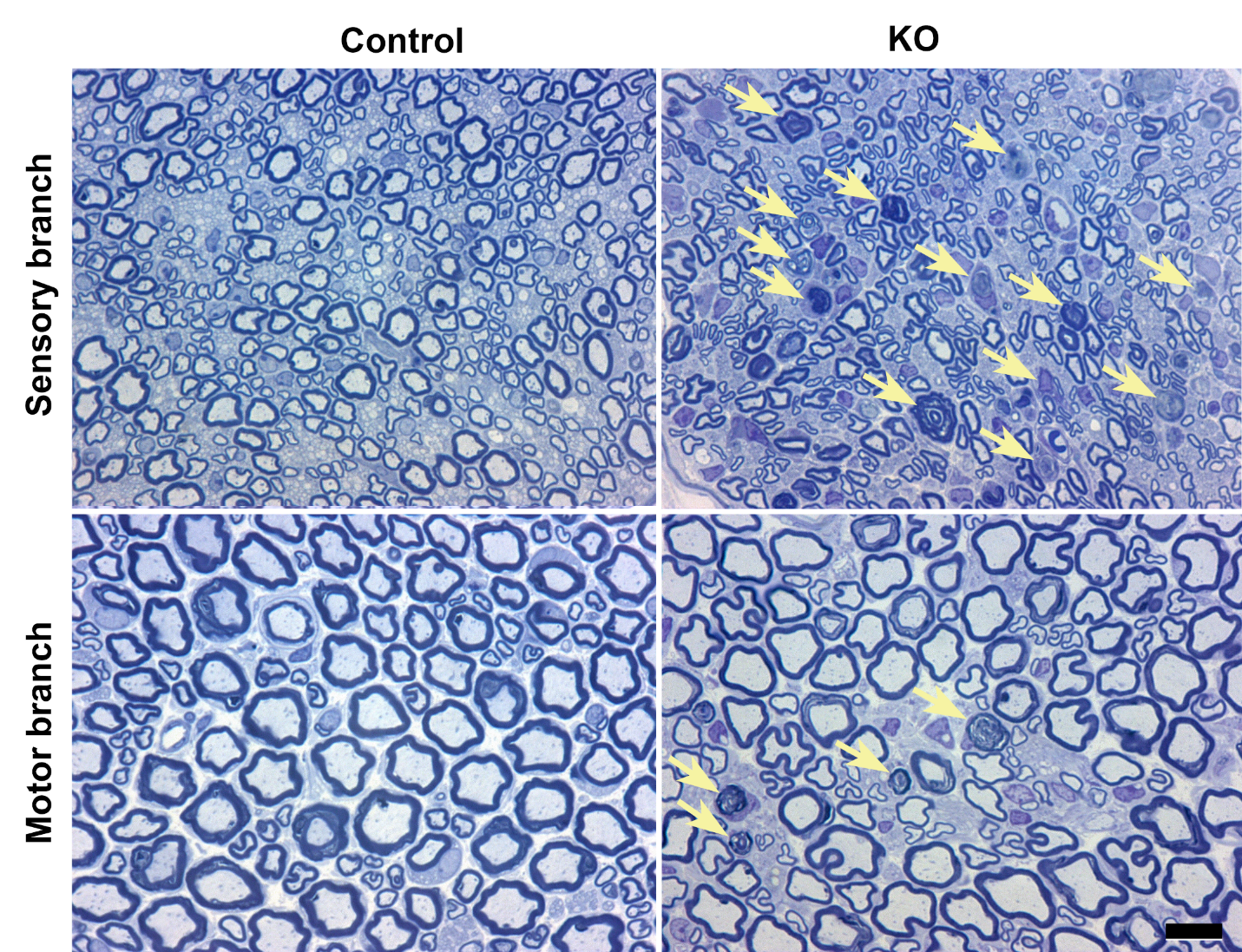
**

**Supplemental Figure 6. Degenerating sensory axons in the femoral nerve of *Clec16a*^ΔUBC^ mice.** These are representative images of semi-thin sections of the sensory and motor branches of a femoral nerve from control/*Clec16a*^loxP/loxP^ and KO/*Clec16a*^ΔUBC^ mice, as indicated by the yellow arrows. Scale bar = 10 μm.

**Supplemental Figure 7.**

**
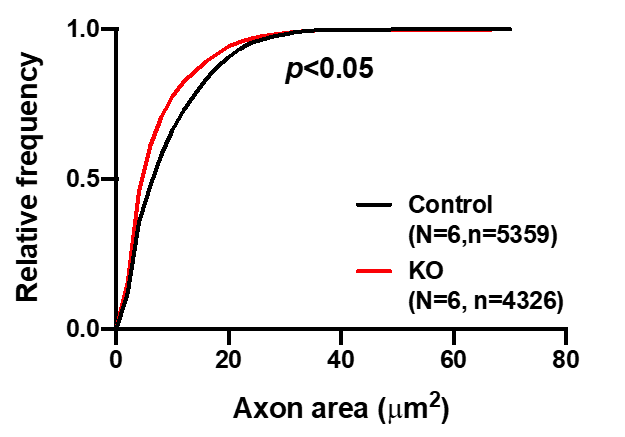
**

**Supplemental Figure 7. *Clec16a*^ΔUBC^ mice exhibit a higher frequency of smaller axons than control mice.** Cumulative histograms of the axon size for femoral sensory neurons from control/*Clec16a*^loxP/loxP^ and KO/*Clec16a*^ΔUBC^ mice. N=number of animals, n=number of myelinated axons counted.

**Supplemental Figure 8.**

**
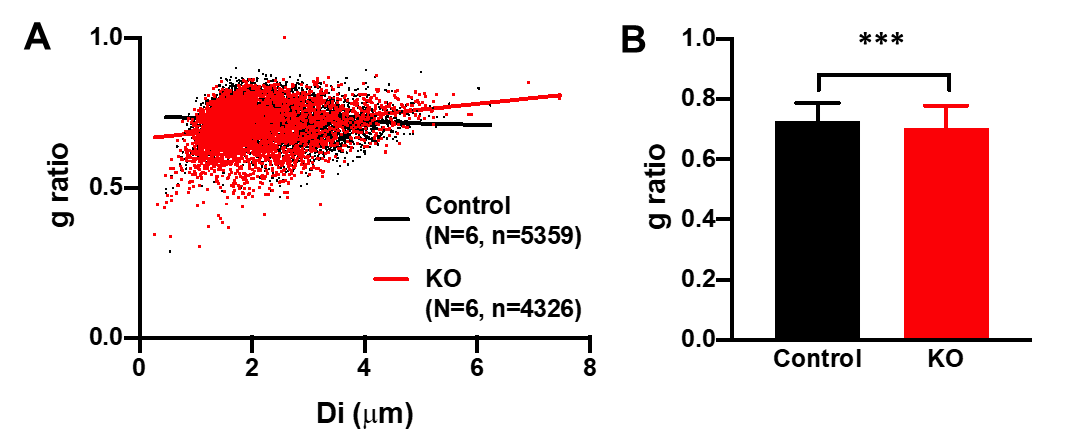
**

**Supplemental Figure 8. Myelin thickness (g ratio) in femoral sensory neurons from *Clec16a***^ΔUBC^ **compared to control** **mice.** To compare the myelin thickness between control and KO groups, the g ratio of femoral sensory nerves from KO/*Clec16a*^ΔUBC^ control/*Clec16a*^loxP/loxP^ and mice were measured. (A) Linear regression results were y=-0.005x + 0.7379 (Control) and y=0.020x + 0.6631 (KO). (B) Mean ± SEM for each group. N=number of animals, n=number of myelinated femoral sensory nerves counted. *** p<0.0001.

**Supplemental Figure 9.**

**
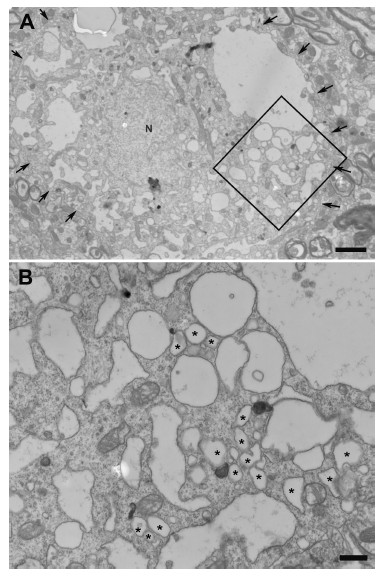
**

**Supplemental Figure 9. Vacuoles in spinal cord neurons of *Clec16a*^ΔUBC^ mice.** These are electron micrographs of the lumbar spinal cord of KO/*Clec16a*^ΔUBC^ mice. Panel (A) shows a large neuron whose cell border is outlined with arrows, containing large cytoplasmic vacuoles. The rectangle is enlarged in panel (B), which shows a cluster of small vacuoles (asterisks). Scale bar = 2 μm (A) and 500 nm (B).

**Supplemental Figure 10.**

**
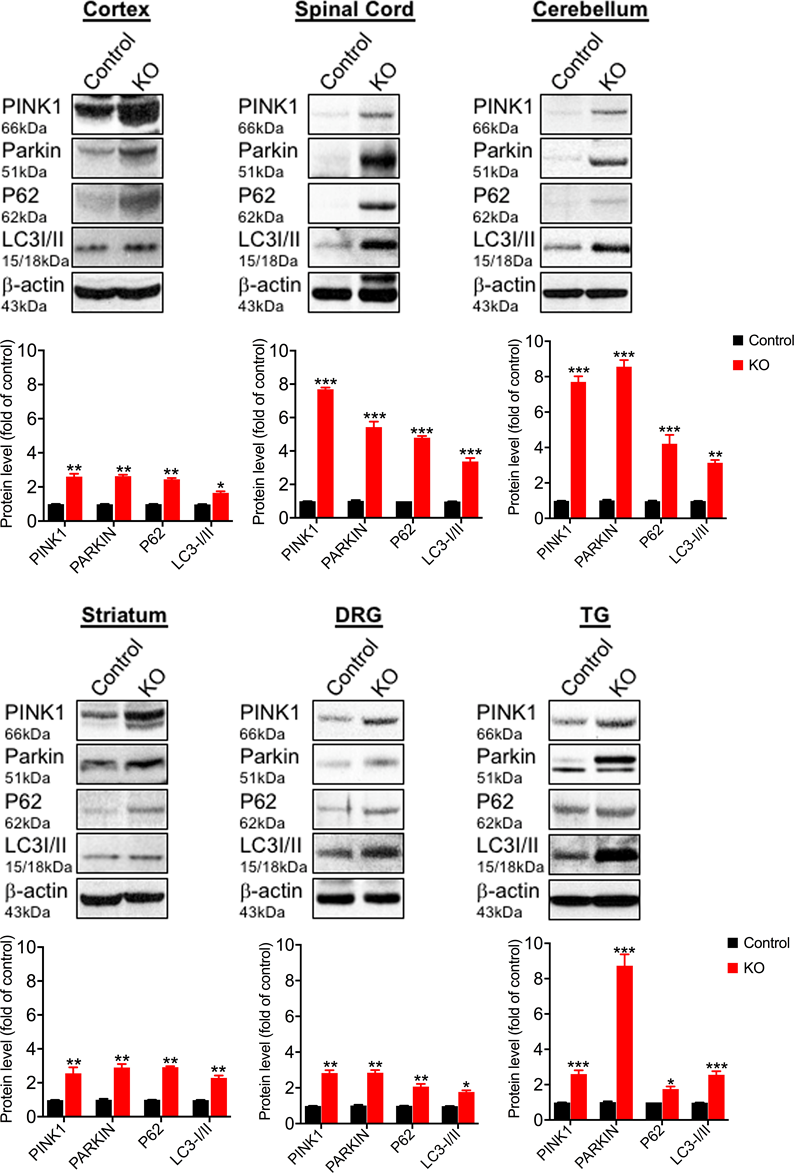
**

**Supplemental Figure 10. Neural tissues from *Clec16a*^ΔUBC^ mice display increased levels of mitophagy proteins.** Representative Western blots show levels of proteins associated with mitophagy in neural tissue lysates from KO/*Clec16a*^ΔUBC^ with severe disability and control/*Clec16a*^loxP/loxP^ mice. β-actin was used as loading control. Quantitation graphs represent mean ± SEM of n=3 samples of each group. *P<0.05, **P<0.01, ***P<0.001 (Control vs. KO) for each protein in each tissue type as analyzed by Student’s t-test. Dorsal root ganglia (DRG), trigeminal ganglia (TG).

**Supplemental Figure 11.**

**Supplemental Figure 11.** **ER stress and OXPHOS markers differences in *Clec16a*^ΔUBC^ DRG and TG.** RT-PCR depicting upregulated ER stress markers in DRG (A) and TG (B) at day 10 and day 22 in KO/*Clec16a*^ΔUBC^ mice as compared to control on day 22 post-tamoxifen administration. (C) Representative immunoblot depicting expression of CHOP in DRG and TG lysates Day 22. (D) Quantitation graph depicting fold change in expression levels of CHOP in DRG and TG. (E) Representative immunoblot depicting mitochondrial OXPHOS respiratory complex protein levels in DRG and TG lysates of KO/*Clec16a*^ΔUBC^ compared to control. A cocktail antibody comprising the following subunits of respiratory complex proteins are used: NADH dehydrogenase (ubiquinone) 1 beta subcomplex 8 (NDUFB8; complex I), succinate dehydrogenase complex, subunit B, iron sulfur (SDHB/Ip; complex II), ubiquinol-cytochrome c reductase core protein II (UQCR2; complex III), cytochrome c oxidase subunit 2 (COXII; complex IV) and ATP synthase 5A (ATP 5A, Complex V). (F) Quantitation graph depicting fold change in expression levels of OXPHOS signaling subunits in DRG and TG. The data was presented as percentage of proteins normalized to porin levels. Membranes were stripped and re-probed for β-actin as a loading control. Data expressed as mean ± SE of three independent repeats. *P<0.05, **P<0.01 ***P<0.001, #P<0.0001 (Control vs. KO).

**Full Blots for Figures**

**Supplemental Figure 13: Full blot images of Figure 7. ISG15 expression KO mice neuronal tissues compared to control.** Representative western

blot for ISG15 expression in cerebellum, cortex, spinal cord striatum, trigeminal and DRG lysates of control, and

KO mice. Membranes were striped and reprobed for b-actin as a loading control.


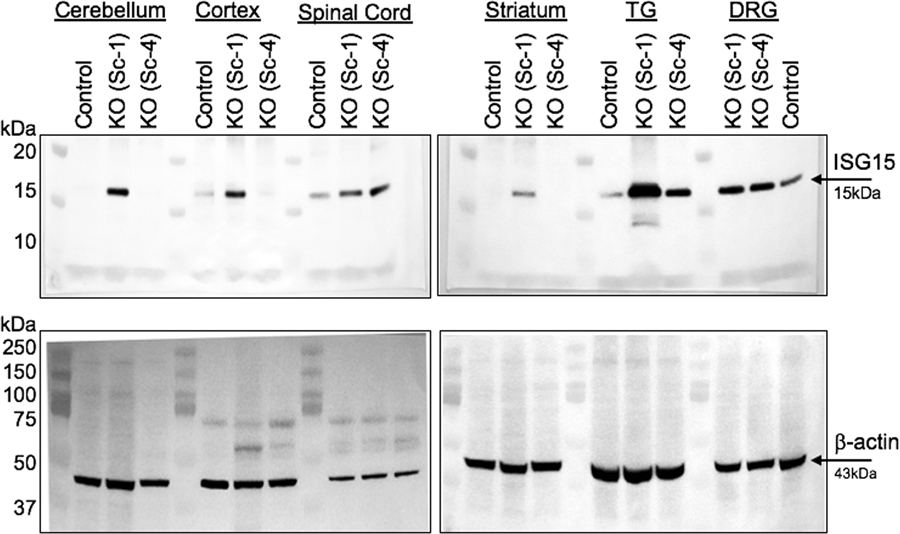


**Supplemental Figure 14: Full blot images of Supplemental Figure 3C**. **CLEC16A expression in control and *Clec16a*^ΔUBC^ mouse tissues.** Representative Western blot depicting CLEC16A expression in whole cortex, hippocampus, cerebellum, spinal cord, dorsal root ganglia (DRG), trigeminal ganglia, and striatum lysates of control/*Clec16a*^loxP/loxP^ and KO/*Clec16a*^ΔUBC^ mice. Membranes were cut in parts and probed for protein of interest where specified. β-actin was used as the loading control.

**Supplemental Figure 14: Full blot Images of Supplemental Figure 10. Neural tissues from *Clec16a*^ΔUBC^ mice display increased levels of mitophagy proteins.** Representative Western blots show levels of proteins associated with mitophagy in neural tissue lysates from KO/*Clec16a*^ΔUBC^ and control/*Clec16a*^loxP/loxP^ mice. Membranes were cut in parts and probed for protein of interest where specified. β-actin was probed as a loading control.

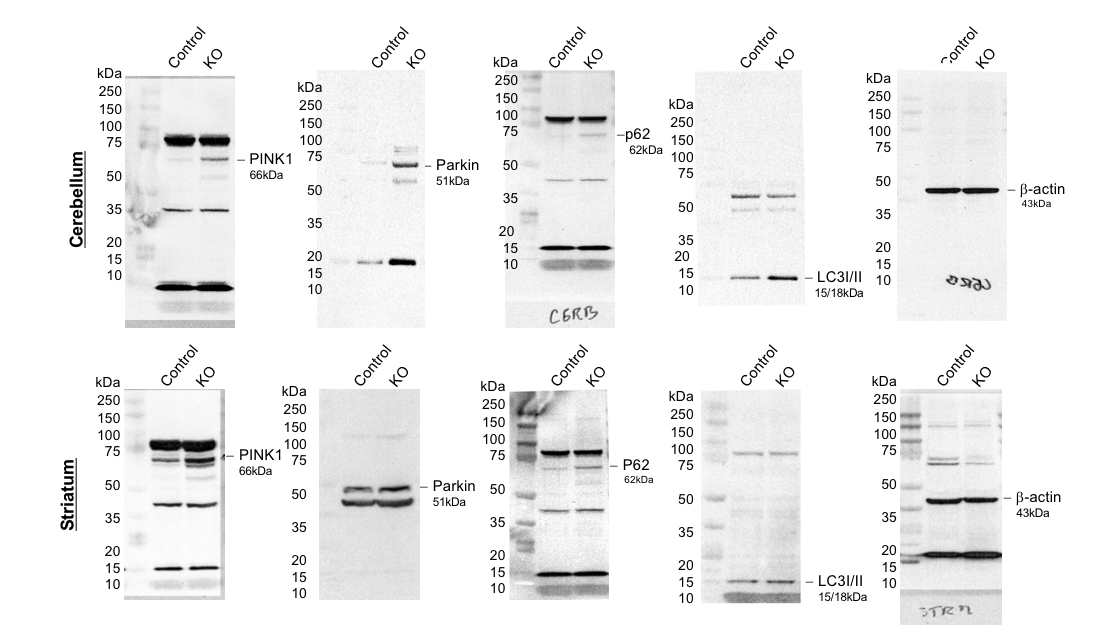

**Supplemental Figure 15: Full blot images of Supplemental Figure 11. ER stress and OXPHOS markers differences in *Clec16a*^ΔUBC^ DRG and TG.**(C) Representative immunoblot depicting expression of CHOP in DRG and TG lysates Day 22. (E) Representative immunoblot depicting mitochondrial OXPHOS respiratory complex protein levels in DRG and TG lysates of KO/*Clec16a*DUBC compared to control. Membranes were cut in parts and probed for protein of interest where specified. β-actin was probed as loading control.
